# Quantitative methods for group bibliotherapy research: a pilot study

**DOI:** 10.12688/wellcomeopenres.17469.2

**Published:** 2024-01-04

**Authors:** Emily T. Troscianko, Emily Holman, James Carney

**Affiliations:** 1 The Oxford Research Centre for the Humanities, University of Oxford, Oxford, UK; 2 Campion Hall, University of Oxford, Oxford, UK; 3 The London Interdisciplinary School, London, UK

**Keywords:** bibliotherapy, evaluation, group reading, narrative, literature, linguistic analysis

## Abstract

**Background:**

Bibliotherapy is under-theorized and under-tested: Its purposes and implementations vary widely, and the idea that ‘reading is good for you’ is often more assumed than demonstrated. One obstacle to developing robust empirical and theoretical foundations for bibliotherapy is the absence of analytical methods capable of providing sensitive yet replicable insights into complex textual material. This pilot study offers a proof-of-concept for new quantitative methods including VAD (valence–arousal–dominance) modelling of emotional variance and doc2vec modelling of linguistic similarity.

**Methods:**

VAD and doc2vec modelling were used on conjunction with qualitative coding to analyse transcripts of reading-group discussions plus the literary texts being discussed, from two reading groups each meeting weekly for six weeks (including 9 participants [5 researchers (3 authors, 2 collaborators), 4 others] in Group 1, and 8 participants [2 authors, 6 others] in Group 2).

**Results:**

In-text–discussion similarity was inversely correlated with emotional volatility in the group discussions (arousal: *r* = -0.25; *p* = ns; dominance: *r* = 0.21; *p* = ns; valence: *r* = -0.28; *p* = ns). Enjoyment or otherwise of the texts was less significant than other factors in shaping the significance and potential benefits of participation. (Texts with unpleasant or disturbing content that strongly shaped subsequent discussions of these texts were still able to sponsor ‘healthy’ discussions of this content.)

**Conclusions:**

Our methods and findings offer for the field of bibliotherapy research both new possibilities for hypotheses to test, and viable ways of testing them. In particular, the use of natural language processing methods and word norm data offer valuable complements to intuitive human judgement and self-report when assessing the impact of literary materials. We also share observations on facilitation protocols, interpretative practices, and how our group reading model differs from other trials of group reading for wellbeing.

## Introduction

It is intuitively plausible that reading ‘literature’ might have effects relevant to mental health and wellbeing, but is it also true? If so, what effects, and by what mechanisms do they arise? Is it possible to generalize, given the vast scope for variation in texts, readers, and contexts of reading?

Research on ‘creative bibliotherapy’ has begun to address these questions. Creative bibliotherapy, the reading of literary texts (which may include prose fiction, poetry, and/or drama) for health benefits, is distinct from ‘poetry therapy’, which tends to use poetry rather than narrative or dramatic forms of literature, and which often includes writing as well as reading poetry. Beyond this, however, *bibliotherapy* is a contentious term, one that encompasses a wide range of practices, contexts, and rationales—from 1-1 encounters in which a ‘bibliotherapist’ analyses a client’s problem and suggests appropriate reading matter to reading groups in which a book is read and discussed during regular meetings. Shared Reading, the most common form of organized reading for wellbeing in the UK and Europe, does not call itself bibliotherapy because any therapeutic effects were initially seen as secondary, with the main goal being to expand access to ‘great writing’: to ‘give immediate access to complex writing that might otherwise be at least daunting and at worst unavailable to a large section of the population’ ^1^. Longden *et al.* ^2^ propose a framing of Shared Reading as ‘implicit psychotherapy’ in which usefulness remains implicit and potential because the activity remains squarely literary (p. 118), concluding ‘We believe that *recovery*, *restoration* or *realisation* may be more appropriate terms than *therapy’* (p. 119). Some studies have, however, made the directly therapeutic potential more explicit, as in Longden and colleagues’ ^3^ investigation of Shared Reading versus waitlist on quality of life for individuals with dementia. Even here, though, ‘wellbeing’ is the reference point and ‘bibliotherapy’ is not mentioned by name.

As in literary studies more generally, only relatively recently has empirical research begun to inquire into the health/wellbeing-relevant mechanisms and effects to individual and shared reading. This research has typically taken a bottom-up data-driven approach rather than adopting the theoretical constructs relied on in much bibliotherapy theory, which consist largely of concepts adapted from psychoanalysis, such as identification and catharsis. Further, when assessing research conducted so far in this broad area, an interesting divergence arises: The majority of existing bibliotherapy theory concerns individual reading, while most empirical work has involved group reading. The theory is based on minimal empirical evidence, and the empirical work has not yet been used to derive a more evidence-based theoretical account, although it has generated many hypotheses for efficacy and mechanisms of change.

Drawing on existing theoretical and empirical research on bibliotherapy, and using relevant tools from other areas (experimental psychology, natural language processing, cognitive literary studies), this study aimed to contribute to the project of mapping out testable hypotheses of bibliotherapeutic change. We adopted a group-reading methodology to connect the theoretically and empirically driven traditions via investigation of both text-centred and broader social aspects of how reading exerts change. In the rest of this introduction, we consider 1) the purported and observed therapeutically relevant effects of creative bibliotherapy in group settings and 2) the hypothesised mechanisms of therapeutically relevant change (in group and individual settings).

### Therapeutically relevant effects

Many wide-ranging claims are made for the therapeutic value of reading, taking in purported benefits to self-understanding, self-expression, and self-esteem; interpersonal and communication skills; and creativity, change, and coping and adaptive functions, amongst others. We acknowledge that we equivocate in our discussion here between ‘therapeutically relevant’ and ‘cognitive and emotional engagement’. However, part of the project of creative bibliotherapy is to purposely challenge any easy distinction between effects of literature that are ‘healing’ and those which foster ‘wellbeing’. It is becoming increasingly the case, for instance, that the literature and practices associated with fostering population-level mental health emphasise wellbeing and self-care as part of health care interventions. Take the ‘Every Mind Matters’ NHS social media campaign. This initiative aims to forestall larger mental health problems by providing audiences with tools that help encourage healthy sleep patterns, avoidance of anxiety triggers, and positive mood. These are not “therapeutically” relevant in the narrow sense but are nevertheless part of the UK government’s focus on improving mental health before it becomes a biomedical issue. Insisting too strongly on a distinction between therapeutic and the salutary effects in reading would, we suggest, close down one of the more progressive innovations in thinking about mental health and wellbeing.

When it comes to putting such claims to the test, the most extensive empirical studies of group bibliotherapy have been carried out by Josie Billington and colleagues in the Centre for Research into Reading, Literature and Society at the University of Liverpool, in collaboration with The Reader. Their interventions typically involve reading a mixture of fiction and poetry, and have included:

- Groups at a GP practice, run by a trained facilitator for patients and local residents ^4^.
- Groups in healthcare settings led by a project worker, for adults with depression ^5^.
- Groups organized for individuals with or vulnerable to mental illness, isolation, or unemployment who volunteer for The Reader plus other local volunteers, led by the founder of The Reader ^2^.
- Groups in a range of community and healthcare settings led by English undergraduates ^6^.
- Groups in prisons led by a trained Reader in Residence ^7, 8^.
- Groups led by a project member in healthcare environments, for people with dementia (and sometimes the staff who care for them), using mostly poetry ^3, 9^.
- Groups for Mersey Care NHS Trust service users, led by a trained Reader in Residence, and also training NHS staff to grow a lasting reading-group culture ^10^.

Billington and colleagues hypothesised that group reading should bring improvement in the areas of social, mental/educational, and emotional/psychological wellbeing, and found qualitative evidence of improvements across all areas, including: enhanced concentration, interest in learning, self-awareness, and capacity for self-expression; increased confidence; reduced sense of isolation ^5^. Similarly, the Mersey Care initiative, assessed in a Merseyside Service User Evaluation, documented ‘improvements in confidence, self-esteem, self-expression, memory, concentration, creativity, social engagement, listening skills and overall health and well-being’ ^10^. Robinson ^4^ reported positive effects on mood, loss of self (being ‘taken out of oneself’), concentration, confidence and self-esteem, pride and achievement, and communication skills, as well as appreciation of the opportunity to reflect on experiences in a supportive environment, and of a common purpose and shared ‘journey’.

Where quantitative measures have been used, reduction in dementia symptom severity ^9^ and improvement on depression markers on the PHQ-9 ^5, 11^ have been observed, though numbers were small and causality cannot be established because neither study included a control group. Other quantitative measures of change are rare, but in a 12-week crossover design, Longden and colleagues ^2^ found a substantial effect size for an increase on the ‘purpose in life’ subscale of the Ryff Scale of Psychological Well-being after 6 weeks of Shared Reading versus a social activity focused on the built environment, where no change or a reduction on this scale was found. No significant differences between conditions were found for other scales administered, including positive and negative affect; depression, anxiety, and stress; mastery; and mental well-being, although some effect sizes suggested trends meriting further investigation. A later study by Longden and colleagues ^3^ found improved quality of life for individuals with mild to moderate dementia with three months of Shared Reading versus waiting control condition, and no change to very low levels of psychopathological symptoms. A systematic review ^12^ found a small to moderate effect on internalizing, externalizing, and prosocial behaviours amongst children from studies with a range of procedures involving stories, poems, or films, plus various forms of interpretive support. A later review of creative bibliotherapy for post-traumatic stress disorder (PTSD) ^13^ found no high-quality studies but did yield some suggestions that understanding and communication may be enhanced by group interventions involving reading. Meanwhile, a study using Persian poetry found evidence of quantitative improvement in mood, specifically a reduction in depression and an increase in hope amongst women with breast cancer receiving chemotherapy ^14^.

In other existing qualitative work, mood improvement is a common focus of inquiry. Mood was treated as the central dimension of change in a qualitative study using poetry about disability, which draws distinctions between *nervous* (i.e. emotional) *arousal*, *energetic arousal* (action readiness), and *hedonistic tone* (valence) ^15^. Pettersson’s user-focused study using poetry and short stories found six main categories of reading function reported by the four reading group participants who completed subsequent interviews: informational, escapist, social, perspective-creational, aesthetic, and therapeutic ^16^. Pettersson points out that the first three of these align with Brewster’s outline of four user-centred models of bibliotherapeutic outcome for mental health problems: informational, escapist, social, and emotional (including empathic and cathartic) ^17^. The absence of the emotional function raises the possibility that, for her participants, the emotional is subsumed in the therapeutic. That said, however, the primary observed benefits were interpersonal and pragmatic, including improved self-confidence and ability to perform simple daily activities (including reading, willingness to engage in social activities, and capacity to complete daily chores). The interpersonal strand of these findings aligns with the characterization of the Shared Reading group as an ‘affordance nest’ for socially distributed meaning-making (and tolerance of the absence of ready meanings) in Skjerdingstad and Tangerås’s ^18^ case study of a single group session.

In sum, then, changes on a wide range of social, cognitive-emotional, and behavioural dimensions are sought and observed in existing literature. Beyond the possibilities that in the absence of controls, randomization, and blinding, researchers are observing what they want to observe and participants are telling researchers what they know they want to hear or what they themselves want to believe, other questions arise. In particular, the breadth of documented effects raises the question of the extent to which they can be attributed to the group meetings or the reading of the text(s). Would a regular group meeting with no literary object of focus, or reading the same texts on one’s own, have similar effects? Is engaging with both text and group contributing something specific? If so, what does each element offer; by what means? Here questions arise regarding mechanisms of change, and there is less evidence to draw on.

### Elicitors and mechanisms of change

What ‘active ingredients’ are responsible for bibliotherapeutic change?

Some researchers draw on existing theoretical frameworks like Vygotsky’s model of deep understanding ^6, 19^ or reader-response models of creative and participatory reading ^5^. Billington and colleagues ^5^ propose ‘four significant components or “mechanisms of action”’: reading material, facilitator, group dynamics, and physical environment. They elaborate as follows:

1. ‘A rich, varied, non-prescriptive diet of serious literature, including a mix of fiction and poetry (the former fostering “relaxation” and “calm”, the latter encouraging focused concentration). Both literary forms allowed participants at once to discover new, and rediscover old and/or forgotten, modes of thought, feeling and experience.’ (p. 6)
2. ‘The role of the group facilitator in expert choice of literature, in making the literature “live” in the room and become accessible to participants through skilful reading aloud, and in sensitively eliciting and guiding discussion of the literature. The facilitator’s social awareness and communicative skills were critical in creating individual confidence and group trust and in putting the group’s needs above those of the individual where necessary. The facilitator’s alert presence in relation to literature, the individual and the dynamics of the group is a complex and crucial element of the intervention.’ (p. 6)
3. ‘The role of the group in offering support and a sense of community.’ (p. 6) (Evidenced by increased ‘reflective mirroring’ of others’ ‘thought and speech habits’, and increased cooperation and personal confidence.)
4. ‘The environment in contributing to atmosphere, group dynamic and expectation of the utility of the reading group.’ (p. 7)

The researchers described the first three as ‘essential in its success’ and the last as ‘influential’. At present, however, these are not falsifiable hypotheses. The observed effects may be due to all, none, or any subset of these factors. The relative importance of the four factors in different iterations may (or may not) also be highly variable between contexts. The individual mechanisms could be to some extent isolated by adjusting reading-group procedures in order to assess their relative contributions—for example, by selecting ‘unserious’ literature, by democratizing the facilitation (as in the present study), or by altering group size and composition or environmental setting.

In the dementia study cited earlier ^9^, brevity and variety of texts, length of meetings (one hour), an informal setting (a lounge), and the presence of a staff member are highlighted as crucial. Robinson ^4^ stresses the importance of reading aloud (as a means of building confidence and sharing encouragement) and of the expert facilitator’s contributions, including in deciding how long to spend informally chatting before reading, judging when to stop reading for discussion, and helping people start with texts accessible and enjoyable enough to stay motivated for the longer term. Focusing more on textual form and content, Daboui and colleagues ^14^ suggest that the spiritual aspects of Persian poetry help in increasing hope, and that poetry as a form generally helps with communication about taboo subjects like death.

These observations of factors contributing to efficacy suggest ways of narrowing down the wide range of possible contributing factors, but they do not lead directly to accounts of the mechanisms by which efficacy is achieved. Several attempts have been made to set out a multistage cognitive process to account for observed changes. Gorelick, for example, sets out four phases of reading-stimulated therapeutic change: recognition, examination, juxtaposition, and application to self ^20^. Billington, Longden, and Robinson ^8^ single out ‘memory and continuities’ and ‘mentalisation’ (exercise of Theory of Mind) as mechanisms by which shared reading may have a protective or therapeutic function for problems such as depression, self-harm, and personality disorders. Montgomery and Maunders propose that the mechanisms of creative bibliotherapy might be roughly equivalent to those of cognitive behavioural therapy: They categorize these into ‘cognitive reading processes’ (recognition and reframing) and ‘emotional reading processes’ (empathy, emotional memories, identification) and suggest that parallel forms of identification, challenging, and replacing of negative thoughts occur in bibliotherapy, resulting in ‘new attitudes and belief systems’ ^12^.

In their later paper, Glavin and Montgomery ^13^ propose that the transporting effect of literary reading may permit a form of exposure therapy in which things that would be threatening in the real world can be safely engaged with in the fictional one. This application of the literary-theoretical concept of transportation brings their paper into contact with theories developed beyond the realm of bibliotherapy specifically, by cognitive literary scholars studying the psychological effects of reading in other contexts of psychological difficulty, including bereavement or post-traumatic distress. Kuiken and Sharma ^21^, for instance, identify the complex phenomenon of ‘sublime disquietude’, resulting from a mixture of perceived emotional discord, self-perceptual depth, and inexpressible realizations, all of which literary reading can induce. Sikora, Kuiken, and Miall ^22^ explore the interplay of presence and absence that occurs during literary reading after bereavement in relation to a gradual acceptance of poignant memories of the dead person. Kuiken, Miall, and Sikora ^23^ investigate how different forms of self-implication affect the emergence of this type of readerly response, especially via the blurring of boundaries between reader and narrator. This in turn relates to broader recent work on the many varieties of ‘personal relevance’ that a text may prompt, with a range of emotional and interpretive consequences ^24^.

Theoretical models from the individual reading paradigm tend to follow a common pattern: They emphasise similarity between the reader’s problematic experience and the arc of the protagonist’s story. This similarity is believed to prompt an identification-based connection between reader and protagonist that generates (possibly via a catharsis-like reaction) insight into the nature of a problem, in turn eliciting a problem-solving phase in which the reader learns from the protagonist and makes personal changes ^25– 29^. This model faces both empirical and theoretical obstacles ^30– 32^, not least a paucity of supporting evidence and a failure to pin down how and to what extent ‘similarity’ is therapeutically beneficial. The limit case here would be reading about one’s own experiences—for example, via diary-writing—and although therapeutic writing has a growing evidence base for many conditions and situations (on poetry therapy, see Ramsey-Wade and Devine’s ^33^ review), it seems unlikely that reading literature written by others should exert its effects via the same mechanisms, merely diluted.

Building on survey data ^32^ suggesting that for at least one type of illness (eating disorders), heightened reader–protagonist similarity can generate reader perceptions of significantly harmful, rather than helpful, effects, in this study we were open to the possibility that reading might generate uncomfortable, distressing, and even apparently anti-therapeutic experiences for readers. We were also open to the possibility that short-term negative experiences, and individuals’ perceptions of them as unhelpful, might not be the whole story; such difficult experiences may contribute to positive longer-term effects. Longden and colleagues ^2^ found that Shared Reading increased negative affect and suggested that rather than being a drawback of the procedure, the effect is ‘consistent with some of its intrinsic value (ie, literature’s power to open individuals up to a range of emotional states)’ (p. 118). This type of hypothesis is compatible with both catharsis and exposure-therapy hypotheses of bibliotherapeutic efficacy, and with a more general view that the value of the experience of literary reading derives from complexity and multidimensionality rather than a simple feel-good effect.

Other perspectives are provided in research showing that readers who score high on a ‘search for meaning’ scale—a metric correlating with propensity for depression—are more receptive to literary (over non-literary) versions of a text ^34^. Similarly, several papers give theoretical and empirical grounds for thinking that fiction can reduce anxiety by offering predictive schemes for thinking about social interactions ^35– 37^. Carney ^38^ also explores the role of predictability in culture more generally, suggesting how entropy (a measure of unpredictability) might differentially impact on anxiety and depression.

## Objectives

The primary objectives of this study were as follows.

1. To record and transcribe a full set of reading-group discussions to generate detailed data on group–text interactions.
2. To trial new computational methods for sensitive analysis of the resulting complex textual material.
3. To generate new hypotheses on potential therapeutic efficacy and its mechanisms to guide future research in group bibliotherapy.

## Methods

### Ethical approval

Ethical approval for this study was provided by the University of Oxford Social Sciences and Humanities Interdivisional Research Ethics Committee, ref. MS-IDREC-C1-2015-155. All researchers involved in this project completed the NIH online training course ‘Protecting Human Research Participants’ and familiarized themselves with the BPS code of conduct and the University’s data protection and academic integrity guidelines.

### Reading group procedures and participants

Our study took the form of two closed ‘Books, Minds, and Bodies’ reading groups in two consecutive university terms (October to December 2015 and January to March 2016), the first group meeting for seven weekly sessions, the second group for eight weekly sessions, as required to complete the selected texts. Two groups were run in order to generate a wider range of text–participant interactions than would have arisen in a single iteration. All 3 authors participated in the first group; EH and ET participated in the second group. The authors took responsibility for welcoming participants, covering housekeeping announcements, and establishing basic guidelines for conduct at the start of the first session, and thereafter participated in the reading and discussion in the same way as all other participants.

Other participants were recruited via an advert posted on noticeboards and local online events listings, offering a chance to ‘explore connections between reading and mental health and wellbeing' with a group of professional researchers interested in these topics. Group 1 included 9 participants (3 authors, 2 colleagues, 4 others); Group 2 included 8 participants (2 authors, 6 others, reducing to 5 others after session 3 after one participant dropped out due to other commitments). Group 1 included 6 females; Group 2 included 7 females, and the male participant was the one who dropped out after session 3, leaving an all-female group. Group 1 consisted solely of students and researchers (ranging in career stage from undergraduate to postdoc), while the four non-author participants in Group 1 were neither students nor researchers/academics. Other demographic information (e.g. age, education, occupation) was not gathered. We sought a minimum of 4 and a maximum of 6 recruited participants per group, to allow for a range of perspectives without compromising the intimacy and trust that can more easily be created in a smaller group. We required participants to be aged 18 years or over, to have an interest in narrative and/or mental health, and to be available for weekly two-hour sessions during the term. Other than age, the only exclusion criterion was that participants be able to confirm that ‘I do not currently suffer from a mental health disorder’. This criterion was included to mitigate the risk of harmful effects being generated by participation. ET interviewed participants beforehand to increase the probability of sustained and positive commitment by providing information to prospective participants about the role they would play in the group. ET provided the information sheet before the meeting, and asking the following questions in person:

1. What drew you to this project?
2. Are you able to commit to weekly sessions until [date]?
3. What are your expectations for the project?
4. Have you ever been part of a fiction reading group?
5. Are you comfortable with a rotating facilitator role?

After the roughly 20-minute meeting, potential participants were invited to take their time to decide whether they wished to take part, and after confirming their interest by email were sent further information on text selection (see below). Paper consent forms were provided for participants to check and sign at the first meeting of each group, with the opportunity to ask further questions. All but one of the interviewed candidates participated; the exception had to pull out due to scheduling difficulties.

Group 1 (henceforth *MT*, for ‘Michaelmas Term’, Oxford University’s autumn term) met at 6 pm on Mondays; Group 2 (henceforth *HT*, for ‘Hilary Term’, the spring term) met at 2:50 pm on Fridays. Meetings took place in one of two similar meeting rooms at Balliol College, Oxford. Texts were selected democratically: We circulated a list of five possible books suitable in length for a term’s meetings, providing either a short (one-page) excerpt or a link to an Amazon.co.uk preview. Text length was the primary factor in shortlist selection; beyond this, we drew on our collective knowledge about texts likely to reward the close attention involved in being read aloud and discussed at length. Each participant ranked the full selection in order of preference and vetoed any book they had read before. Fyodor Dostoevsky’s *Notes from the Underground* was selected for MT (in the Oxford World Classics translation by Jane Kentish) and Ted Chiang’s *Story of Your Life and Other Stories* for HT. We chose not to read the same text in both groups to ensure that all participants shared in the experience of discovering a new text together and also to capture as much variance as efficiently as possible (rather than attempting to control for variance as will be appropriate in a follow-up study). 

We also note in passing that issues of ‘literariness’ and ‘quality’ emerge as an item of concern here, given that creative bibliotherapy centres on the idea that literary works are the drivers of therapeutic and cognitive change. On this question, we had to strike a balance between identifying texts that no one had read and honouring individual preferences. The two resulting texts are works of recognised quality, in that one is by a major European novelist (Dostoevsky) and one is by a leading contemporary practitioner of science fiction (Chiang). We suggest that these choices withstand critique on grounds of literary achievement, and that the distinction is, in any event, an unhelpful one: What counts as literature has always been contested, and what counts as a diversion in one historical period can become the literature of a subsequent one.

Participants were presented with their own copy of the selected book, provided with a pencil, and encouraged to make markings in the book if they wished. Copies were collected between sessions to ensure participants did not read ahead on their own; these were given to participants to keep at the end of the term. In the final MT meeting, having finished reading the main text, we asked each participant to bring a poem of their choice to read and introduce to the group. In the final two HT meetings, having read four Chiang stories, we read two short stories by Franz Kafka in English translation: ‘A Hunger Artist’ and ‘Jackals and Arabs’. These additional sessions are excluded from the linguistic analysis of the literary texts and the transcripts (six for each term) covered in this article.

During the sessions, we opted to invite all participants to contribute equally from the outset to reading the text aloud. In some Shared Reading studies, the authors observe that participants grow more confident and co-operative over time: Billington and colleagues (2010) ^5^, for example, note that over the 12 months for which the group met, participants increasingly ‘took the initiative in supporting one another’s comments, in guiding the direction for discussion and in offering to read aloud from the text themselves’ (p. 7; see also Robinson, 2008, pp. 6–7 ^4^). Here we invited such co-operative contributions from the outset. Books were read aloud by participants, switching with each paragraph (or, where these ran for longer than a page, with each new page). Sessions lasted 2 hours, the first hour spent reading, followed by an hour for discussion with refreshments (wine / soft drinks and nibbles in MT, tea and biscuits in HT).

Audio recording began at the start of the second hour, and no notes were taken in addition to the recording. In another contrast with the typical protocols for Shared Reading, we chose to adopt a shared facilitation model in which each session was facilitated by a different participant, usually self-nominated. This decision was taken for two reasons: 1) to allow the groups to be conducted and participated in by ourselves as a research team with no training in formal facilitation in Shared Reading or any other model; 2) to reduce any perceived hierarchy between the researcher participants and others (whether academics themselves or otherwise).

Facilitation was described to participants in the facilitator information sheet as follows:

The facilitator role, which will rotate at each session between participants, is broadly to keep the conversation going. This involves making sure that all participants feel able to contribute and perhaps occasionally asking someone who has been speaking for a long time to open the conversation to others. No more than one person should be speaking at once—and no private comments or conversations. This is a group discussion.

We have put together a list of possible questions that might be useful when you are in this role.

The questions included 5–7 questions in each of the following 4 categories:

- Emotional response (e.g. ‘Do you care what happens to the main character(s)?’)
- Interpretive response (e.g. ‘Did you ever feel that what was being described wasn’t what the passage was really “about”?’)
- Mental imagery (e.g. ‘Do you find you’re imagining more or less or differently than you do when you read alone?’)
- Drawing connections between real life and the narrative (e.g. ‘Do you think anything in this passage could help you think through or deal with difficulties in your life?’)

The questions were designed to cover a wide range of potential types of response to the texts, going beyond interpretive, meaning-focused responses to encompass emotional and sensory dimensions, as well as opening up possibilities for connections with real-world experience that might be relevant to health and wellbeing. In practice, the questions were barely used, since conversational prompts from facilitators were rarely needed, and facilitators in general preferred to generate them ad lib when required.

Collectively, the decisions to democratize the choice of text to be read, the reading-aloud of the text, and the group facilitation meant that our procedures deviated significantly from the Shared Reading tradition, in which a trained ‘reader leader’ selects the texts, reads it (though contributions from others may be invited), and facilitates the discussion of it. Together they maximized the self-contained simplicity of the group-reading setup: there was no prior training based on complex pre-existing theory or tradition. They also maximized the democratic nature of the experience, given that all participants played comparable roles in the selection, the reading, and the guiding of discussion.

A final difference from the Shared Reading protocol was that the reading of the text proceeded uninterrupted (aside from brief clarifications of vocabulary or similar) for a full hour. This format allowed us to record the entirety of the discussions without needing to record the reading as well; this was useful given that some participants expressed a degree of nervousness or inhibition about reading aloud to begin with. The clear demarcation also allowed the direct and indirect experiences of textual engagement to unfold with their own distinct dynamics, rather than the reading being repeatedly interrupted by discursive reflection. This allowed for greater analytical clarity. Overall, our priority was to create a simple set of group-reading procedures in which to trial quantitative methods for assessment of the group’s practices and effects, rather than to either replicate or challenge Shared Reading or any other model.

After both groups had concluded, EH and ET transcribed the audio recordings of the discussions, EH taking MT and ET transcribing HT. One HT session was transcribed by a group participant, who was paid for her contribution; her transcript was checked and edited. Participants’ contributions were pseudonymized using colour codes to introduce their discussion interventions, and the codes were stored separately from the transcripts in a password-protected file.

While aware of the potential for researcher bias in a participatory framework of this kind, we considered that it could not be directly minimized at the group participation stage given the naturalistic setting, but that variations in individual perspectives would be manifest by all participants (some of whom were, in MT, also academics in other fields, i.e. there was no simple dissociation between ‘researchers’ and ‘participants’). In contrast to many prior studies, all forms of linguistically manifested bias were made explicit in the discussion recordings and transcripts, and were thus treated as an integral part of the complex dynamics under investigation. At the stage of analysis as opposed to participation, meanwhile, our methods were expressly designed to reduce the problematic forms of bias evidenced in qualitative content analysis, as we go on to document below.

***Participant feedback*.** After the final reading-group session, participants completed an online survey designed to complement our analysis of their discussion contributions with direct self-report on the experience of taking part. The survey included questions about the enjoyment and significance of different elements of the reading-group experience, and forms of learning and change participants perceived to have resulted from taking part. To test for differences between the two groups, feedback from participants was analysed using an independent samples t-test that compared means between the two groups.

### Comparison with other studies’ procedures

The procedures used in our study both resemble and differ from other studies along several dimensions. Table 1 summarises the comparisons.

**Table 1.** Comparison of procedures across cognate studies.

	Clinical participants	Poetry/ mixed genres	Participants co-choose text	Topic- led reading	Trained facilitator	Discussions recorded	Reading aloud by participants
Our study (BMB)	N	N	Y	N	N	Y	Y
Robinson (2008)	N	Y	N	N	Y	Y	Y
Billington *et al*. (2010)	Y	Y	Y	N	Y	Y	Y
Robinson & Billington (2012) ^39^ / Billington, Longden, & Robinson (2016)	Y	Y	N	N	Y	N (but interviews with staff recorded)	Y
Longden *et al.* (2015) ^2^	N	Y	N	N	Y	Y	Not specified
Longden *et al.* (2016) ^3^	Y	Y (poetry only)	N	N	Y	N	N
Tukhareli (2011) ^40^	Y	Y	N	Y	Y	N	N
Pettersson (2018)	N	Y	N	N	Y	N (but post-group interviews recorded)	Y
Dubrasky *et al.* (2019) ^41^	Y	Y	N	N	Y	N	Y (shared equally)
Daboui *et al.* (2018)	Y	Y	N	N	Y	N	Not specified
COMMENTS on the present study		Last session of MT involved poetry	Researcher team created a shortlist				Shared equally amongst participants

### Analytical methods

All analysis as outlined below was conducted on the full dataset resulting from 17 total individual participation instances (this included 2 repeat participations by researchers EH and ET) minus 1 participant who dropped out for non-study-related reasons after session 3 of HT.

***Rationale*.** The guiding principle of our data analysis was that the discussion transcripts, including their linguistic relationship to the texts under discussion, stand at the centre. This analytical principle derives from the hypothesis that if shared literary reading is having wellbeing-relevant effects, they will be visible in the linguistic patterns of the text-prompted discussion. We acknowledge that the inclusion of control and experimental groups would represent the optimal way to evaluate the effects of interest here. However, our aim was not to engage in formal hypothesis testing, but to gain pilot data from which hypotheses could constructed. With respect to existing research in this area, some previous studies have recorded and transcribed discussions ^2, 4^, but transcripts have been relatively underused as sources of analytical insight. Our methods focused on two dimensions of participant response: emotional reaction and cognitive elaboration. Reading is a complex amalgam of emotional and cognitive responsiveness, and any thorough appraisal of its processes and impacts needs to attend to both ^42^. As our ambitions included quantifying the reactions of our reader groups, this meant finding ways to measure the cognitive and emotional variation implicit in our data. We resolved this problem by making use of word norm data and unsupervised machine learning in the following ways.

***Emotional response*.** Traditionally, an impediment to supplementing qualitative assessments of the impact of reading has been the difficulty of measuring subjective responses. This is especially so with respect to emotional response, given that there is no universally attested taxonomy of emotion. When this emotional response is expressed verbally, the facility of language for expressing the same emotions in different ways compounds the problem. It follows that quantitative analysis of our transcript data has to try to solve the problem of extracting nuanced emotional information from text.

Our response to this challenge was to make use of word norm data. Essentially, word norms are corpora of language that have been rated by human participants along a specific dimension or set of dimensions ^43– 45^. Warriner and colleagues ^44^ present 13,914 common English lemmas (words stripped of morphological variation) that have been rated by human participants for *valence*, *arousal*, and *dominance*. According to dimensional models of emotion, each discrete emotion can be represented in terms of an underlying set of finite components ^46^. The VAD model of affect identifies valence, arousal, and dominance as the underlying factors responsible for emotional variation ^47, 48^. That is, each discrete emotion can be thought of as a specific combination of valence (how pleasant or unpleasant it is), arousal (how stimulating or sedating it is), and dominance (how in-control or controlled it makes someone feel). Thus, anger is a low-valence, high-arousal, low-dominance emotion, while contentment is high-valence, low-arousal, and high-dominance. These extensive word-norm data provide an empirically validated way of assessing the overall emotional impact of a word, making mean VAD easy to calculate. Though valence is often understood in polar terms (positive or negative), we followed Warriner and colleagues’ (2013) ^44^ approach of treating it as a scalar metric (0 = low valence, 1 = high valence) to allow for comparison with arousal and dominance, which are not polar in nature. We could have performed a median split to capture polarity, but we felt it best to retain the original formulation in Warriner *et al.*’s data for the same reasons as they offer. The great value of the VAD norms is that they provide a low-dimensional proxy for emotional variation that is not restricted to words that are ostensibly emotional in their character (mood words like ‘happy’, ‘sad’, ‘angry’, etc.). They therefore provide a versatile means of establishing emotional variation on the basis of word use in linguistic documents. To this extent, they have an obvious value when it comes to capturing how our participants responded to texts and to each other on a per-session basis. Necessarily, they are also limited: emotion is conveyed not just by lexical choice, but also by phrasing, tone, and body language; equally, as averages of rater responses, VAD word norms are not sensitive to homonymy and other subtleties of usage. Moreover, competing dimensional models exists, with different dimensions as well different numbers of dimensions ^46^. No doubt, these other dimensional models of emotion could have been used if word-norm data existed on a similar scale to VAD, and this could have affected our results. However, we are confident that that the alignment of the VAD dimensions with physiologically and socially fundamental features of human emotional cognition means that any differences would be in detail and terminology, rather than in our broad conclusions. In any event, certainty on this point could only be secured only by re-running the analysis using word-norm data that do not presently exist.

The emotion of each text (both the discussion transcripts and the literary texts under discussion) was calculated by taking the mean of valence, arousal, and dominance across all the words of that text. Although this has the advantage of computational simplicity, within-text random variation means that the longer the text, the more they will tend towards the background mean for these values for English. As our texts were relatively short and of roughly equal length, we felt that any effects of reversion to the mean would be small and spread equally across all texts. This analysis was performed by JC using bespoke scripts written in python 3; the script ascertained the value for each word on the transcript for each session for valence, arousal, and dominance using the Warriner ^44^ data and returned an average for the entire session. With respect to taking the mean of responses as our variable of interest, we acknowledge that a case could also be made for using the variance: Literary scholarship is, after all, at least as interested in the role of texts in stimulating variation in responses as in coordinating them. However, as our study was guided by the assumption that there would be coordinating effects of reading texts in groups, any subsequent statistical testing would reveal if there wasn’t by not allowing us to disqualify the null hypothesis that texts have no coordinating effects on responses. As the use of variances would not have been of value in generating testable hypotheses consistent with our aims, we therefore did not use them as point estimates of our data.

***Cognitive elaboration*.** Perhaps a greater challenge than measuring emotional response is measuring cognitive response. Allowing that the difference between ‘emotional’ and ‘cognitive’ is somewhat artificial, the fact remains that the space of possible concepts is wider than the set of possible emotions—it is, in fact, at least the size of a language’s vocabulary. Given that concepts can also be recursively combined to produce new concepts, this means that the set of possible concepts is combinatorically large. Until recently, this has meant that the task of extracting topics and themes from linguistic documents has been the preserve of the best pattern-matcher we know: the human brain. However, advances in unsupervised machine learning mean that it is now possible to automatically identify the specific ways in which words are combined with others to produce recurring items of content. In particular, word-embedding algorithms like word2vec, doc2vec, GloVe, and BERT provide empirically robust methods for capturing semantic variation at the level of word, document, and context that can be deployed at scale ^49– 53^. Like all machine-learning methods, these algorithms are sensitive to initial parameter selection, so there is no sense in which they provide objective measures of semantic variation. Nevertheless, they inject a useful amount of statistical rigour into a practice that is often a hostage to ideological agendas.

For our purposes, the algorithm of most value was doc2vec, a document-level analogue to word2vec. Where word2vec represents the behaviour of a word across a corpus of documents by training a shallow neural network to predict its association patterns, doc2vec is trained by associating document tags with the word vectors comprising the document. (These word vectors are mathematical descriptions of how a word behaves in a corpus; the tags are the document name used to group the word vectors associated with a specific document.) Doc2vec thus represents high-level semantic variation across documents in a precise way, thereby making the discrete documents of a corpus comparable. In our case, the relevant corpora consisted of the transcripts of each session and the relevant portions of each text read in that session. The specific implementation of the doc2vec algorithm we used was that associated with the Gensim natural language processing library for python ^54^. We note here that the doc2vec algorithm operates by representing a document as a mathematical vector and using this representation to (amongst other things) compute document similarity; it does *not* capture specific forms of textual identification. If readers, for example, identify with characters in the discussion and talk about it, then this may impact on the doc2vec metrics. However, there is no way of establishing this, and it was not our intention to use doc2vec to do this.

**Figure 1.**
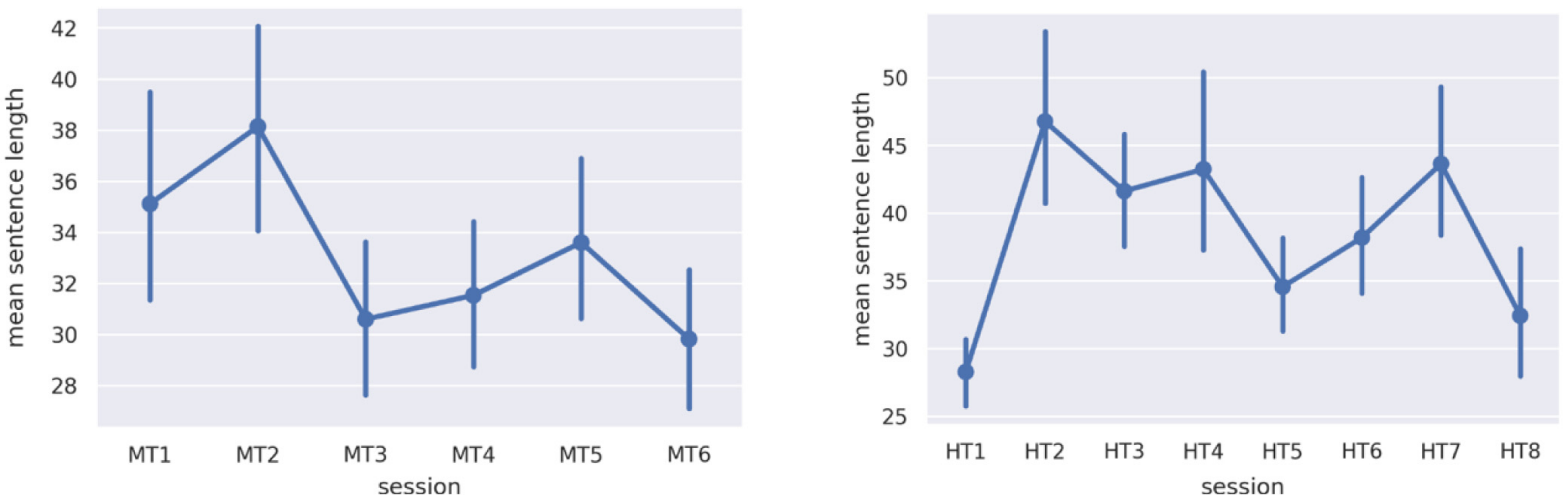
Average sentence length per group for discussions in **a**) MT and **b**) HT. MT=Michaelmas Term; HT=Hilary Term.

When using the doc2vec algorithm, the key parameters involve 1) choosing the size of the moving window of words amongst which semantic relations are assumed to obtain and 2) specifying the number of training epochs used by the algorithm. The first parameter is essentially a discourse specifier: In genres like poetry, this window should be large, given the dense interdependence of semantic elements; in technical writing it should be small, in view of the emphasis on strict denotation. In our case, we resolved on using the average sentence length per group ( Figure 1), given that sentence lengths in discursive conversation can often be quite long. The number of training epochs was 30k, which trial and error show is the point at which results stabilized.

***Data cleaning*.** Both emotional and cognitive measures required texts to be cleaned in a way that made them amenable to automated analysis. In practice, this meant tokenizing the text into words and phrases and eliminating redundant variation across these words and phrases. Tokens were extracted by splitting character strings on whitespace; these were regularized using parsers from the spaCy natural language processing library for python ^55^. In practice, this involved lemmatizing each token, removing case variation, eliminating punctuation markers, and dropping stopwords (‘it’, ‘a’, ‘an’, ‘on’, ‘the’, etc.) that conveyed no semantic information. This reduced each text to a list of words that captured its semantic content in a consistent way.

***Qualitative coding*.** EH and ET read the full set of transcripts and conducted a manual coding process for relevant features. Given our interest in the potential of group reading to have impacts on life beyond reading itself, and given existing theoretical work founded on notions of ‘similarity’, we adopted the categories of ‘personal relevance’ outlined by Kuzmičová and Balint ^24^ as our starting point. These included: *personal relevance*, *perceived similarity* / *simile-like identification*, *wishful identification*, and *metaphor-like identification*. Other categories identified in the first 2 transcripts from each term as necessary to the coding process included: *expression of emotional engagement*, *expression of no emotional engagement*, *engagement with character*, *liking* (of text), *dislike* (of text), *self-qualification*, and *human condition*. The resulting 11 categories were applied to coding the remaining transcripts.

## Results

### Emotion by session

**Figure 2.**
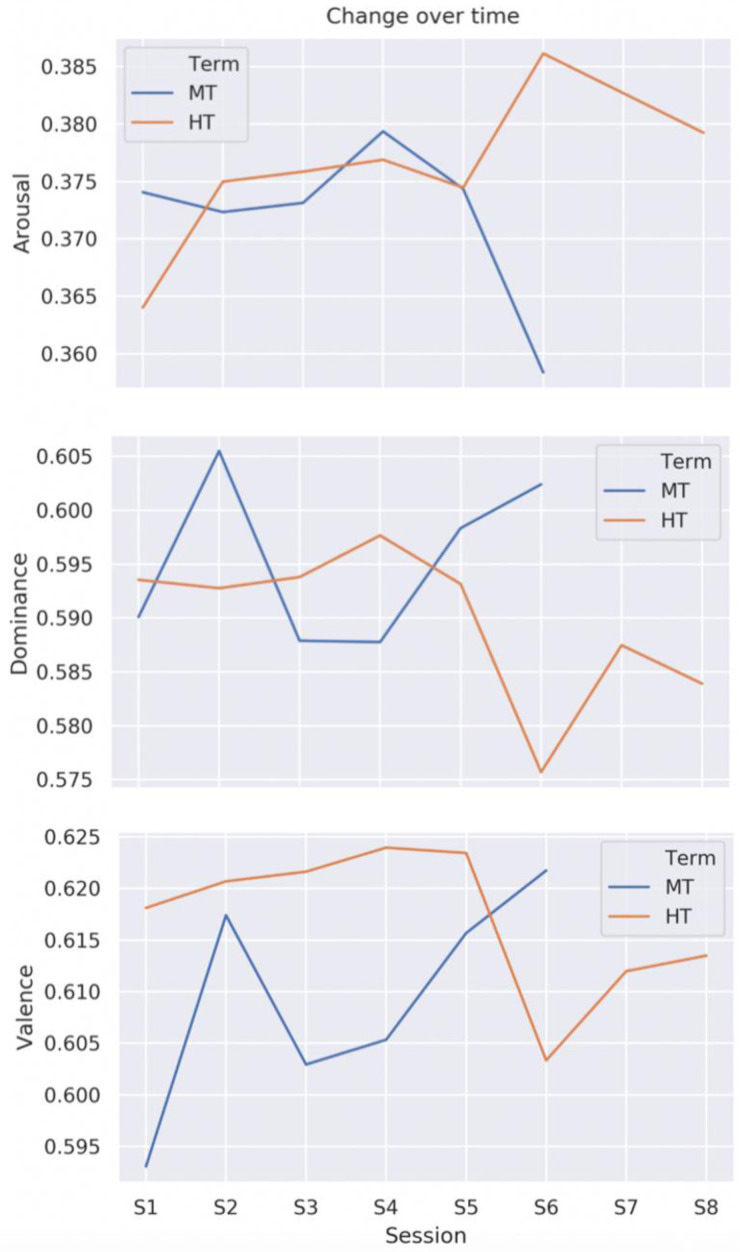
Change in levels of arousal, dominance, and valence by group and session. MT=Michaelmas Term; HT=Hilary Term. The numbers on the y axis are scaled between 0 (min.) and 1 (max.).

VAD analysis of the transcripts showed clear differences in emotional profile both between individual sessions and between the two groups ( Figure 2) ^56^. On the whole, HT exhibited higher levels of valence and arousal and lower levels of dominance across sessions, though these differences were not statistically significant ( *V*: *t* = -1.52, *p* = .16; *A*: *t* = -1.3, *p* = .21; *D*: *t* = -1.38, *p* = .19). This may point to greater emotional febrility in HT, but it may also be an artefact of discussing very different texts. Although the lack of significance indicates that the differences may be the result of random chance, linguistic data exhibit high variation, meaning that significance is unlikely to be found in a sample size this small whether an effect is present or not. We therefore proceeded to analyse the emotional profile of the texts under discussion.

We found that text–discussion similarity was inversely correlated with emotional volatility in the group discussions (arousal: r = -0.25; *p* = ns; dominance: *r* = 0.21; *p* = ns; valence: *r* = -0.28; *p* = ns). In practice this means the greater the level of similarity between texts and discussions as measured by the doc2vec algorithm, the lower the arousal in the discussion of the text. That is, people used less energizing or stimulating language––a quality associated with emotionally volatile language. This possibly arises from there being less disagreement amongst individuals concerning their reception of the text. (See additional data in the online repository: text_sim.)

It should be noted that MT-S6 had no text as such; instead, participants were invited to reflect on their experiences of participation and their relevance for further investigation of bibliotherapy. This meta-reflective structure presumably accounts for the outlier low arousal value for this session relative to the others. In Table 2 we offer a brief indication of some possible reasons for the outlier status of the HT-S6 VAD values.

**Table 2.** HT, Session 6 versus other HT sessions.

HT, Session 6		
VAD	Value (values scaled from 0 to 1)	Discussion features
**Valence**	Term average *M* = 0.616; *SD* = 0.153 Session average: *M* = 0.609; *SD* = 0.153	• Discussion of skin problems, disfigurement, and anxiety about looks. • Topics related to negative aspects of religion, e.g. extremism, sociocultural tension, gender roles, shame, guilt, and hell/damnation. • Discussion of mental health issues, e.g. suicide and addiction in family contexts.
**Arousal**	Term average: *M* = 0.381; *SD* = 0.101 Session average: *M* = 0.390; *SD* = 0.101	• Inflammatory topics (religion and female attractiveness in contemporary culture). • Frequent lack of direct textual connection.
**Dominance**	Term average: *M* = 0.594; *SD* = 0.109 Session average: *M* = 0.577; *SD* = 0.106	• Frequent interruptions. • Discussion of female oppression by men and/or religion.

### Emotion by literary text

**Figure 3.**
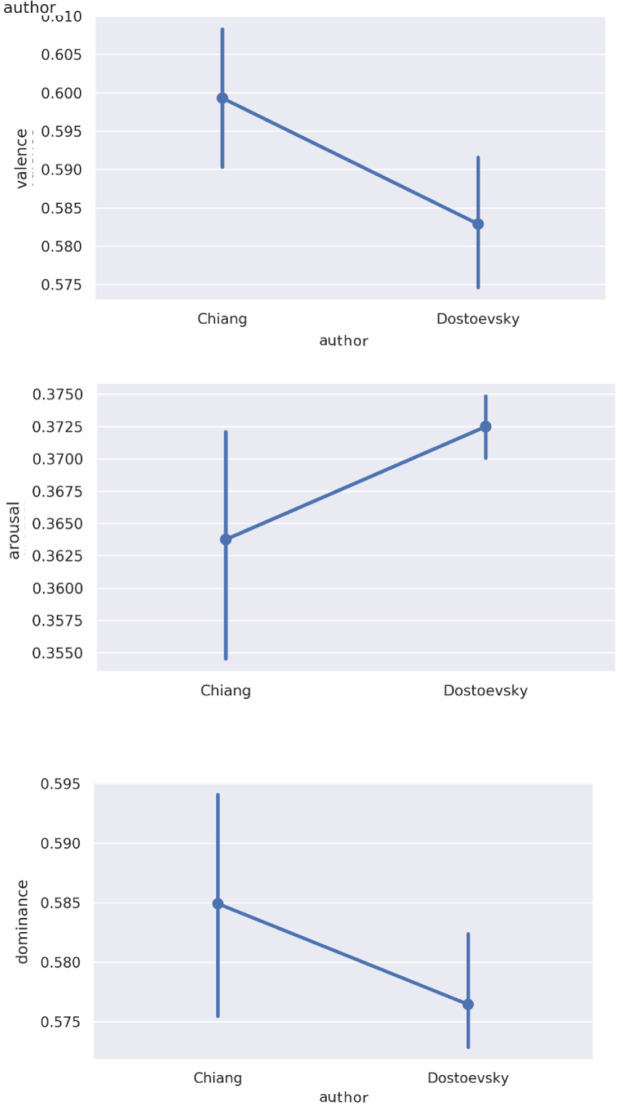
Average **a**) valence, **b**) arousal, and **c**) dominance values for Ted Chiang’s *Story of Your Life* and Other Stories and Fyodor Dostoevsky’s *Notes from the Underground*.

**Figure 4.**
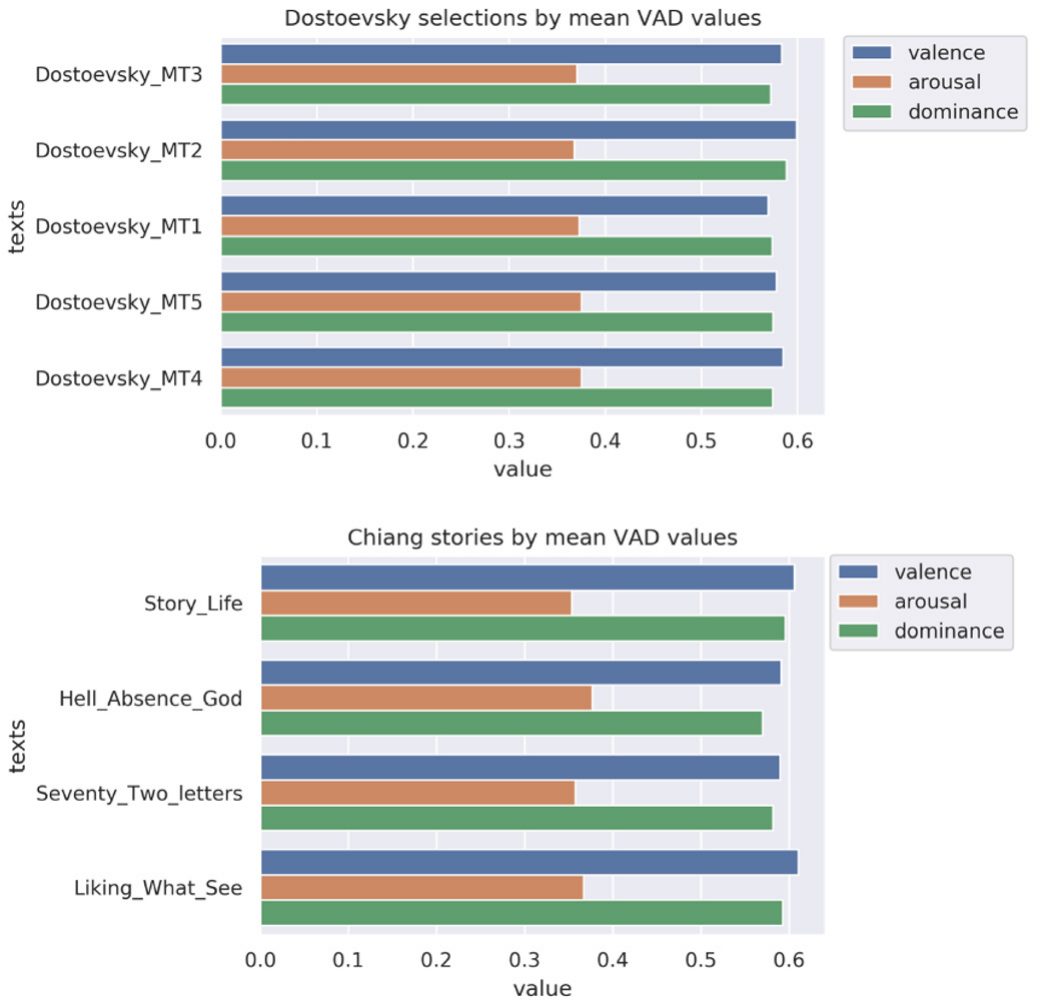
VAD (valence, arousal, dominance) values in **a**) Dostoevsky and **b**) Chiang by session selection.

Since one possible driver of emotional responsiveness in participants is the emotional profile of the texts they are discussing, we took VAD measures of the discussed texts. Considered in aggregate, these measures positioned Chiang as on average lower in arousal and higher in valence and dominance than Dostoevsky ( Figure 3). These differences were not statistically significant; nor did we expect them to be, given likely reversion to the mean for both authors over longer stretches of text. Within each author, there were relatively small differences between specific sections or stories ( Figure 4).

### Emotion by word norm data

**Figure 5.**
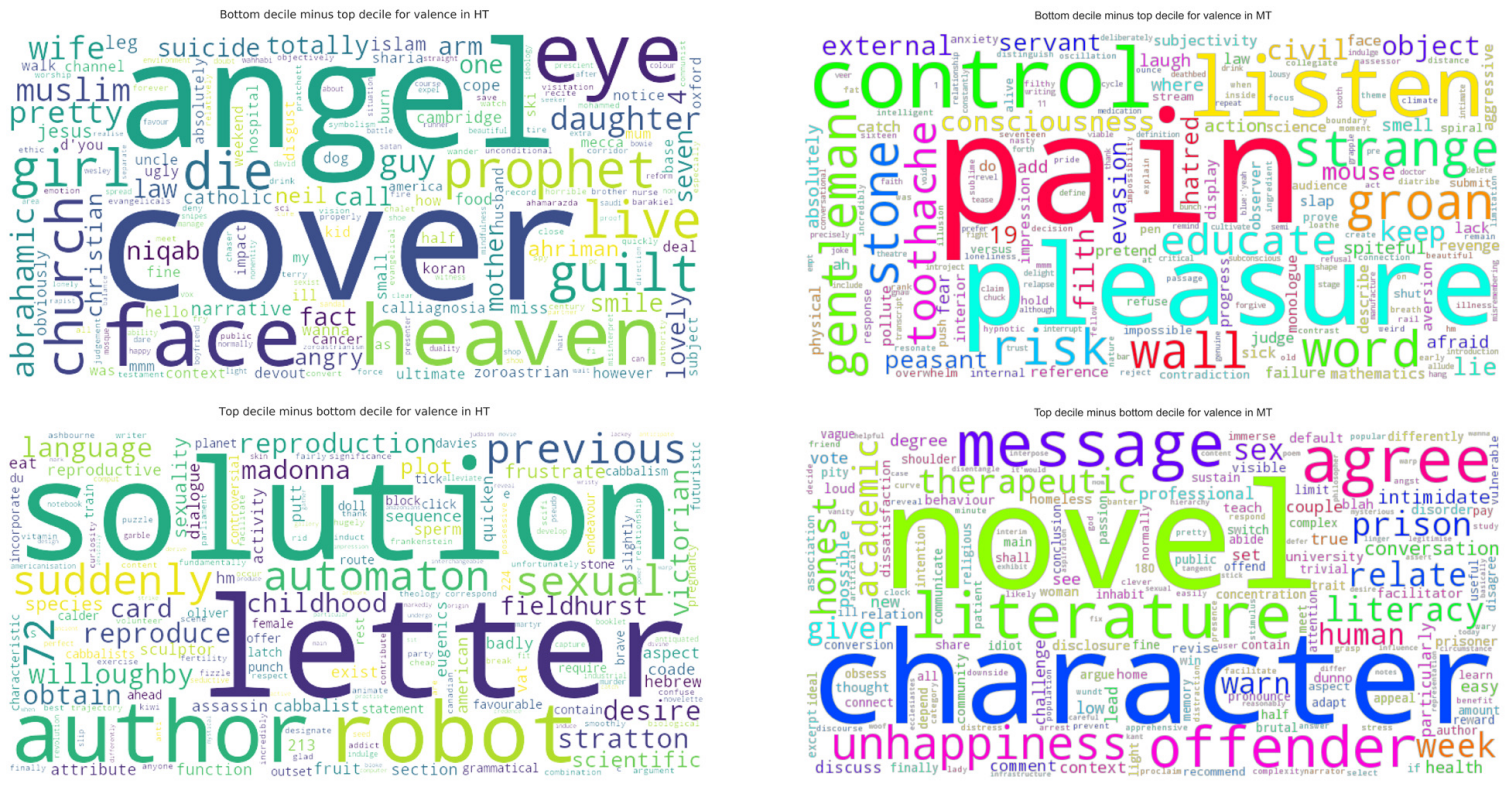
Sample word clouds for valence in MT and HT. The top row shows the words most responsible for negative valence in each term, the bottom row those most responsible for positive valence. Because most words have some positive valence (the scale goes from 0 to 1), words common to both lists were excluded. MT=Michaelmas Term; HT=Hilary Term.

An important element of establishing emotional variation involves identifying the actual words used. We did this by concatenating all transcripts for each group so as to create two corpora. After establishing VAD values for each word, we performed a decile split for each of valence, arousal, and dominance so as to split the data into ranked tiers. By comparing the top and the bottom deciles, this allowed us to gain insight into the kinds of word driving the emotional profile of each group (see an example in Figure 5).

### Cognitive elaboration

**Figure 6.**
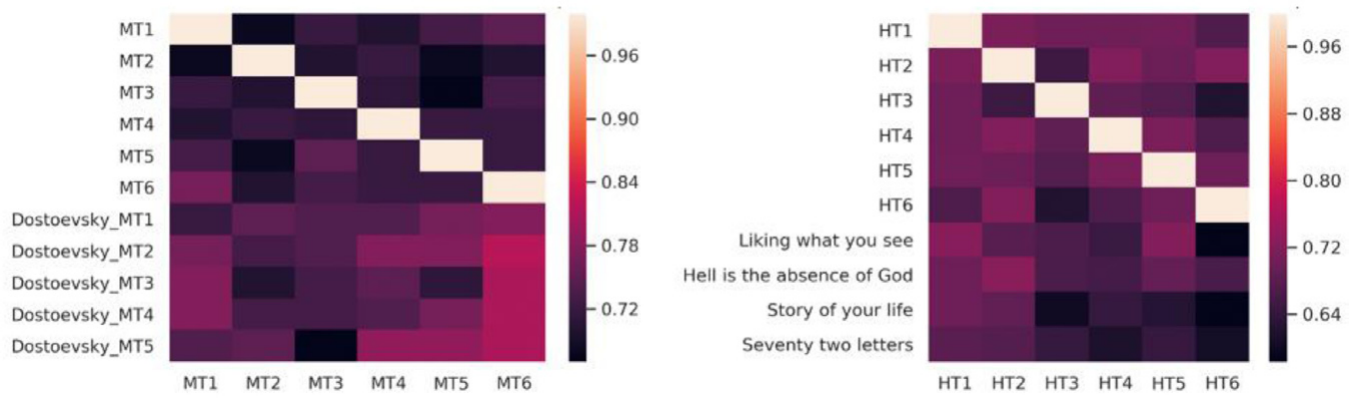
Semantic similarity between transcripts and texts in MT and HT. Note that a value of 0 means purely random association and a value of 1 means identity. Thus, lighter hue means more similar semantic content. Here, this is visible in the bottom half of the left-hand figure, which shows that transcripts were more similar to Dostoevsky selections than to each other. Note that, unlike in MT, HT transcripts in aggregate semantically resemble each other more than they resemble the Chiang texts. MT=Michaelmas Term; HT=Hilary Term.

Our expectation was that measuring doc2vec document similarity between transcripts and texts for each group would show up a pair-wise relationship, such that the transcript of a session would be semantically closest to the text read in that session. Surprisingly, this was not the case: there was no obvious pattern linking a text to a session, and the method was, in any event, highly sensitive to parameter selection. What did emerge were striking between-group differences with respect to whether the group reproduced literary content in general, relative to non-literary content (i.e. the degree to which transcripts resembled the texts being read or resembled other transcripts) ( Figure 6). As can be seen, the MT discussions reproduced far more of the semantic content of the Dostoevsky selections considered in aggregate than the HT sessions with the Chiang stories. In other words, the MT group was more ‘on topic’ than the HT group, if being ‘on topic’ is taken to mean discussing the texts. An additional finding was that in sessions where the doc2vec similarity was low, VAD ratings varied more, suggesting that these sessions were more emotionally febrile.

### Participant feedback

**Figure 7.**
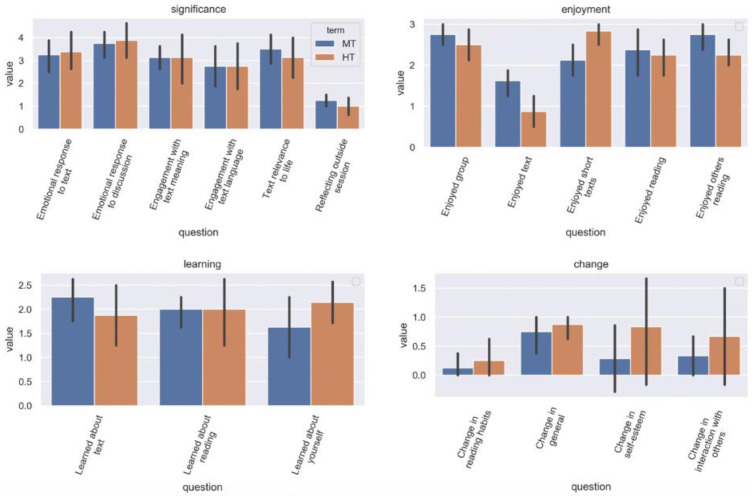
Differences in post-participation feedback, by group and theme.

In both groups, most responses to the post-participation questionnaire followed a similar pattern ( Figure 7). In assessing the significance of different elements of their reading and reflection during the sessions, the participants rated highest their emotional responses to the text and discussion, plus the perceived relevance of the texts to their lives. Engagement with the language of the text was rated lowest; engagement with textual meaning was rated of intermediate significance. Participants rated enjoyment of all elements of participation (the group, the additional short texts, reading aloud, listening to others read) significantly higher than enjoyment of the main text. Learning (about the text, about reading, and about oneself) was rated relatively low, and change (in reading habits, self-esteem, interactions with others, and in general) was rated as low (with general change higher than more specific types).

**Figure 8.**
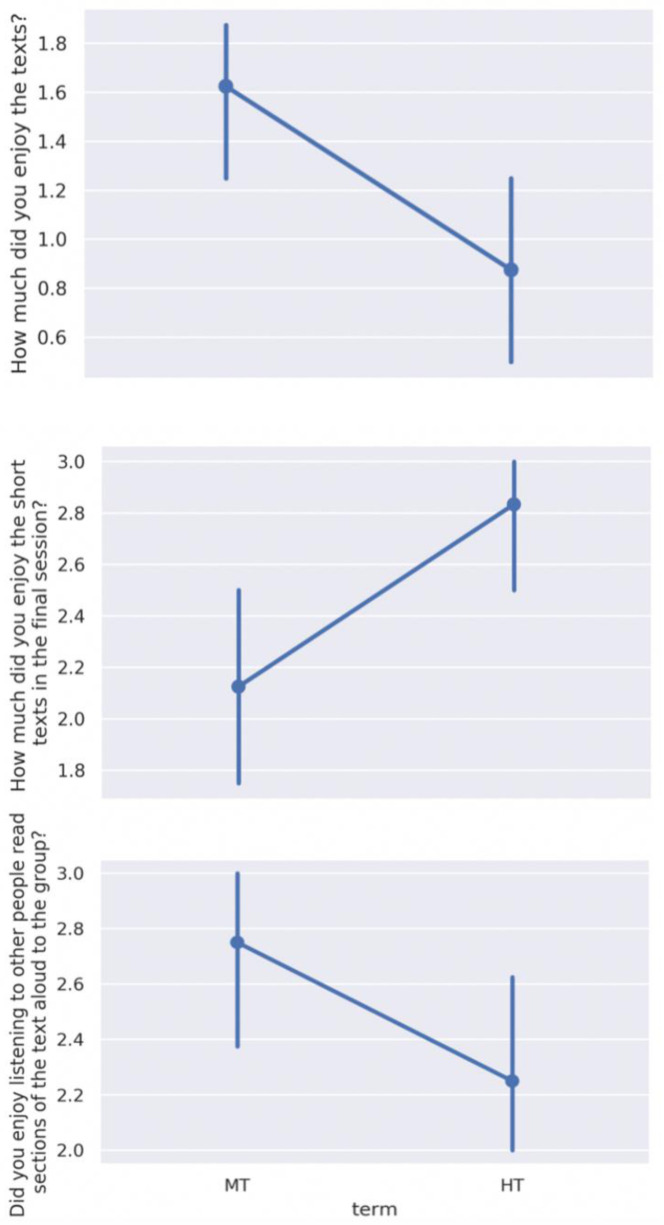
Significant intergroup differences in post-participation feedback: enjoyment of the principal texts, enjoyment of the short texts, and enjoyment of hearing others read aloud. Scales range from 0 to 5, where a higher score indicates greater agreement with the statement. MT=Michaelmas Term; HT=Hilary Term.

Intergroup analysis of participant feedback revealed three statistically significant differences amongst the 32 questions posed ( Figure 8): 

- How much did you enjoy *Notes from the Underground* / *Story of Your Life and Others*? ( *p* = .02)
- How much did you enjoy the short texts in the final session? ( *p* = .03)
- Did you enjoy listening to other people read sections of the text aloud to the group? ( *p* = .04)

### Qualitative coding

For the full coding results, please see the online repository (qualitative_coding_results). Five categories (expression of emotional engagement with the text; self-qualification [no. of instances]; self-qualification [no. of words]; and liking and dislike of the text) were considered relevant and coded by both EH and ET; 3 categories (engagement with a character, expression of complexity or difficulty, and personal revelation) were coded by EH only; and 6 (explicit absence of emotional engagement, personal relevance, perceived similarity, metaphor-like identification, wishful identification, and human condition) by ET only. Differences were manifest between our coding outputs for the 5 common categories; we felt that these were in theory resolvable, but not relevant to the purposes of the present analysis. ET’s use of the categories derived from Kuzmičová and Balint ^24^ resulted in near-zero outputs for ‘metaphor-like identification’ and ‘wishful identification’ (1 instance across the 2 categories in all 12 transcripts), low levels of perceived similarity (21 total instances), and medium levels of personal relevance (68 instances). ET’s self-generated category ‘human condition’ (expressions of commonalities in human experience) yielded a far higher total (120 instances), with significantly more in MT than HT (82 v 38).

### Liking and disliking the text

We found that enjoyment and dislike of the texts read did not impact on how valuable participants felt the sessions to be. Statistical analysis of post-participation feedback indicated that reported enjoyment of the main texts being read was low, and significantly lower for HT than MT. Even when texts were explicitly disliked, participants enjoyed the discussions they prompted, and all other aspects of their participation. One HT participant remarked in response to the question ‘How much did you enjoy the stories by Ted Chiang?’, ‘ *It did not matter. The stories, even when not enjoyable, still triggered discussions, interactions and people expressed their opinions*.’ Other aspects of enjoyment may play an important role: for instance, reported enjoyment levels for listening to the texts being read aloud by others were higher overall, and significantly higher for MT than for HT, supporting the relative insignificance of enjoyment of the text itself in overall experience and effects of participation.

### Liking and disliking the discussion

Many discussions of emotional register focus on whether the register is positive or negative—that is, on valence. We found that valence alone was insufficient to capture the range of emotional variation in how participants reacted to the sessions. Valence levels were higher in HT discussions. However, HT also manifested higher levels of arousal (associated with stress) and generated reports of finding the space unsafe. Conversely, MT participants evinced lower valence and arousal but higher dominance. In both terms, however, participants reported low levels of change in self-esteem or social interaction.

## Discussion

In this study, we designed a novel variant on group reading for wellbeing, differentiated from commonly used Shared Reading protocols in that all participants contributed equally to choosing which text to read, to reading the text aloud, and to facilitating the discussion. These design choices aimed to democratize the group reading experience and to provide a test intervention not associated from the outset with a complex set of assumptions and training protocols as in the Shared Reading methodology. In these senses, the procedures capitalize on the group setting as an opportunity for exertion of personal agency in a social context—aligning with Skjerdingstad and Tangerås’s ^18^ emphasis on the group’s potential to drive distinctive forms of distributed cognition. These choices increase the contrast with the directed nature of the individual bibliotherapy model, where the bibliotherapist selects the text, the patient/client reads the text alone, and the discussion is guided by the therapist.

Our analysis used new quantitative methods in the attempt to provide a combination of richness and replicability needed to answer questions about human responses to complex aesthetic phenomena. Using VAD (valence–arousal–dominance) modelling of emotional variance and doc2vec modelling of linguistic similarity to analyse the discussion transcripts and texts under discussion from two reading groups, we found an inverse correlation between text–discussion similarity and emotional volatility in the group discussions. Specifically, doc2vec analysis demonstrated that in verbal similarity taken as a whole, MT manifested significantly higher levels than HT, but that high arousal plus low dominance were also present. We also found no link between discussion valence and therapeutically relevant outcomes: The higher-valence group discussion (in HT) involved higher arousal and lower dominance, and neither group reported appreciable change in self-esteem or social interaction attitudes/habits. This suggests that higher valence does not necessarily translate into outcomes that reflect autonomy and self-directed action—traits that are often absent in mental health conditions.

Post-participation feedback also suggested that enjoyment or otherwise of both the texts and the discussion was less significant than other factors in shaping how participants perceived the significance and potential benefits of their participation. This suggests that any therapeutic use of fiction need not be restricted to straightforwardly enjoyable or accessible texts. Given that all participants contributed to text selection, and that this selection was presumably guided at least in part by anticipated liking or enjoyment (though perhaps also by emotional responses like identification), the low levels of enjoyment are striking. Anecdotally, it seemed that the opening pages of the chosen texts were considerably more straightforwardly enjoyable than later material. However, other potential selection aids (book blurbs, excerpts from later in the text) would come with their own drawbacks. The finding that enjoyment of the text is not paramount aligns with Shared Reading’s common emphasis on elements of reading that are not dependent on or reduced to enjoyment. Skjerdingstad and Tangerås ^18^, for instance, suggest that the group dynamic may make enjoyment less relevant than it is in individual reading by allowing for intrinsically shared emotional-interpretive experiences such as being moved by another group member’s emotional reactions or personal disclosures in response to the text. It contrasts, however, with other suggestions that a minimum level of enjoyment is important to achieve: ‘However it was important that the groups enjoy what they are reading, or at least want to continue with the book’ (p. 9) ^4^.

Our findings are limited by the absence of a direct control group in which either the same participants read a different book or new participants read the same book. We decided against this option in order to allow everyone to experience a new text together and to maximize rather than control for variance. Any follow-up research should involve a control condition to establish causal links between textual features and discussion variables. Further limitations emerge with respect to our not having captured factors of interpersonal variation to do with reading history, education levels, or personality type. As these data may have mediated the impact of the texts read, including them in future research may provide more material for hypothesis generation and testing.

Analytically, our ability to draw robust conclusions from the linguistic data was limited by the absence of both a strong signal and a large sample size (of discussion material plus texts under discussion). This limitation could be addressed by rolling out such group meetings on a larger scale and pooling the textual materials, although automated transcription tools would in this case ideally be trialled to reduce the time investment of manual transcription. Other expansions such as testing effects of texts in non-narrative genres, or involving participants with specific demographic characteristics or (with appropriate safeguards) with current mental health conditions, could serve to evaluate the generalizability of the current findings.

Our results are based on a small sample of individuals in an idiosyncratic study setup. As such, caution should be used when generalizing to larger samples of readers. However, almost all real-world reading occurs in idiosyncratic ways, and by running the two groups we sought to control for some of the relevant variation between individuals. Moreover, it is hard to see how any experimental setup concerning group reading can capture the large number of factors that attend group reading. For these reasons, we suggest that some aspects of our results may generalize, but further research will be needed to establish which.

In the remainder of this section, we offer some starting points for broader interpretation of our findings with respect to the types of text–discussion interactions that may be therapeutically positive, as well as timescales of potential change. We begin by linking our work with other researchers’ observations on group facilitation procedures.

### Reading group facilitation

The role of the reading group facilitator has been the subject of frequent discussion in existing group bibliotherapy research, and the standard approach is to use a trained ‘reader leader’ rather than to share facilitator responsibilities amongst participants. The experiences of ET and EH in this study suggest there may be benefits to rotating facilitation to involve all participants actively in steering the group dynamics; the key, however, is that facilitation occur, and that it be active. Active facilitation is important in any scenario involving structured discussion of literature, for several reasons. In the first instance, it keeps discussion focused on the text. This aligns with Billington’s reflections on the facilitator’s role, which emphasise the specifically literary guidance facilitators give: one ‘essential’ component for ‘success’ is ‘The role of the group facilitator in expert choice of literature, in making the literature “live” in the room and become accessible to participants through skilful reading aloud, and in sensitively eliciting and guiding discussion of the literature’ (p. 6) ^5^. In the second, the facilitator role regulates conversational dynamics between participants. This supports Robinson’s observation that facilitator duties include ‘bring[ing] people in as much as possible into the general discussion’, being ‘willing to give everyone space to talk and read and reflect, and being ‘able to make them feel that their contributions to any discussions was [sic] valuable and interesting to others in the group’ (p. 5) ^4^. Finally, there is a value in having an individual present who can respond to unanticipated or problematic issues emerging over the course of the discussions, and minimize any potential damage—always a possibility when reading complex texts that elicit a wide range of experiences. All of this becomes more important when the group contains researcher–participants, because they may be inclined to take a more passive role in in their capacity as observers. ET’s personal reports after HT sessions, for instance, highlight the difficulties of participating, facilitating, and researching all at once: ‘ *I tried to “facilitate” and thereby prevented myself participating.*’ The researcher perspective is encapsulated in ET’s habit of ‘ *reminding myself that this is all part of the experiment, and that it doesn’t matter how it goes; it matters that we learn something from how it goes.*’ Sometimes more active intervention is needed, whether by a pre-trained facilitator or from a group member who assumes this role with lighter-touch guidance—perhaps emphasising the importance of encouraging proximity to the text. The choice of facilitation protocols will depend on who is taking part and for what reasons.

### Open and closed interpretation

What types of text encourage constructive interpretive and discursive patterns? Our experience was that texts that were interpretively ‘open’ did a better job of sustaining discussion and promoting a sense of shared purpose than texts that were interpretively ‘closed’. The former are texts that allow readers freedom to speculate as to their meaning without having an obviously ‘right’ answer; the latter are more like puzzles that can be solved in a singular, unequivocal way. The distinction may be aligned with Barthes’ ^57^ distinction between *lisible* (readerly, or literally readable) and *scriptible* (writerly, or literally writable) texts: the former directing interpretation down well-worn paths, the latter drawing attention to themselves and inviting interpretive elaboration. Our result converges with the less specific suggestion made by Billington and colleagues ^5^ that one of the four mechanisms of bibliotherapeutic action is that the literature being read (a mixture of fiction and poetry) be ‘serious’ and ‘rich’, although it may contradict the suggestion that the fiction ought to foster ‘relaxation’ and ‘calm’. Responses to open (or closed) complexity may be beneficial without being relaxing or calming.

Another discovery was that interpretive openness in a text facilitated deeper social interactions between participants than are typically had between strangers. Participants reported that hearing interpretive contributions from others generated curiosity and that later this was rewarded by discovering the personal origins of the contribution:

*they’d come up and have an opinion on some part of the chapter that we read, and then I’d think ooh why did they think that, you know, that’s really mysterious [laughter] And then over the next couple of sessions I’d learn more about them, and that was really interesting.* (MT-S6)

However, this positive effect is lost when discussion strays too far from the text. We should also note that change in broader social interactions was not widely reported in the post-participation feedback, perhaps because of the nature or duration of this intervention relative to the patterns of participants’ everyday interactions.

### Text–discussion proximity

Direct correspondences between text and discussion seem not to be manifested in the emotional sphere in either group. However, our direct probing of text–discussion similarity via the doc2vec analysis demonstrated a clear difference between the two groups: In verbal similarity taken as a whole, MT manifested significantly higher levels than HT. For HT, the highest level of text–discussion similarity occurred when ‘Liking what you see’ was under discussion, a story about physical appearance, social appraisal, and other topics that have a bearing on day-to-day social life. This was the only story that participants said (in discussion and in the post-participation feedback) they had thought and talked about outside the session, and one participant described it (near the start of the subsequent session) as having ‘ *probably resonated with me more than the others, just I guess cos it’s kind of more immediately applicable to everyday experience*’ (HT-S6). Correspondingly, participants in both groups considered the act of drawing connections between the text and their own lives to be of relatively high significance to their experience of reading and reflecting during the sessions. One mechanism of its significance may simply be that by definition it encourages text–discussion proximity, and therefore a greater experience of control during the discussion.

Our results for both groups (an association between low doc2vec similarity and high VAD variance) suggest that discussion needs to be grounded in the interpretive possibilities of the text for it to be therapeutically positive. There is, however, no direct connection on VAD grounds between the emotional profile of the texts and the discussions. Our results therefore challenge the similarity hypothesis concerning bibliotherapeutic mechanisms, in which benefits are derived via a close pedagogical relationship between the protagonist’s psychological situation and progression and the reader’s. We found that value for personal insight and wellbeing was sometimes derived despite the lack of obvious markers of similarity between reader and protagonist. For instance, the Underground Man’s lack of growth was perceived by this MT participant as a spur to personal growth:

*Particularly after the first few sessions in terms of how people occupy such different headspaces, and also discussions about him wanting to control social situations and set up a moment where he is in a certain position of power/standing shoulder to shoulder. It made me think more about not controlling relationships around me, which was helpful!* (post-participation feedback)

When asking how text–discussion similarity is maintained or lost, the difference between discussions centred on author intention versus character motivation seems instructive. The former tended to deflate the world of the text and have the effect of closing down interpretive activity by assuming that ‘definitive’ answers exist. The latter kept the text interpretively open and thereby stimulated ongoing discussion, perhaps because there is clearly no singular ‘fact of the matter’ when it comes to a fictional character’s motivations. One linguistic manifestation of this difference across the two groups was that the pronoun ‘he’ referred more often to the author in HT (along with ‘they’, also for the author) and more often to the protagonist in MT; engagement with characters was also correspondingly higher in MT (see online data file qualitative_coding_results). This challenges the suggestion made by Robinson that author and character are equivalent as objects of interpretive engagement: that ‘participants appeared to become enmeshed in the plot, as they developed their own theories around characters’ actions and motivations, and the author’s intent in using particular words, or including descriptions of particular contexts’ ^4^. In our experience, speculation about the author may be more likely to lead to closed discursive forms: statements of liking/dislike, or statements aimed at establishing biographical facts.

### Human condition, self-qualification, and VAD-guided word frequency as indicators or mediators of positive group dynamics

One important mediator of positive discussion dynamics may be a category that emerged in the qualitative coding of the discussion transcripts: reflections on the human condition. This emerged in a bottom-up fashion out of the attempt to employ the four major categories of ‘personal relevance’ set out by Kuzmičová and Balint, and the realization that none of those categories accommodated an important and frequently recurring phenomenon: the act of seeing something in the text or character(s) as resonating with a general or universal human tendency, or of seeing a character as an ‘everyman’ figure, etc. For instance:

GOLD: *I really loved the um, the bit about memory. [pause] Again because I think it’s true. There are things in everybody’s memory that he doesn’t divulge to everyone but only to friends and so on. I thought that was… [pause] And that writing is often the way of processing those things in a preparatory way, to revealing something. [pause] There’s no self apart from an autobiography in some sense.* (MT-S2)

GREEN: *He’s also got that typical outsider syndrome, of feeling that you’re superior to everybody else, and looking down on them, while also feeling extremely insecure when he’s actually in their presence, so that you’re living constantly in this world of your own making.* (MT-S3)

Frequency of *human condition* mentionings was significantly higher in MT than in HT (though low in the outlier S6 for both groups), and sessions that included most mentions also tended to include higher numbers of expressions of emotional engagement. It is possible that this type of personal relevance-drawing serves as a happy medium between making and expressing a direct personal connection and keeping discussion at an unthreatening but perhaps also ineffectual level of generality. The group reading context in particular may encourage this form of less individualized relevance attribution, as a way of speaking for oneself but also for a collective. Such comments may have a beneficially inclusive effect, and may also involve cause-and/or-effect links, or ambiguous overlap, with more directly personal connection-making. In this exchange, for instance, ORANGE’s human condition mention generates BLUE’s personal relevance mention:

ORANGE: *Because um—let me have a look. [pause] Sorry, I just have to just check back to the things I underlined. [pause] Because he’s obsessed with his social footing and the status, but he tries to achieve it by bumping into people or—the way people look at him, and there’s this constant sense of shame and—he talks about things like physicality and physical size, and I just found it really sort of—a bit like the primate rank, fighting. In a way that I don’t feel—or I don’t—I haven’t experienced or thought about as much in the sense of like the female gender. I don’t know. I think that’s why.*

BLUE: *I think I was probably just instantly translating from kind of jostling with shoulders to I don’t know, idiots not getting out of the way of me when I’m on my bike or something, and then size of body to like shape of body, or... I think I was probably doing the switch very automatically and that was why it didn’t feel alien.* (MT-S3)

The danger also exists that expressing a personal view on a universal phenomenon may make others feel excluded or misunderstood by unjustified generalization. Specifically, the capacious pronoun *you* may sometimes serve as a linguistic veil for a contribution arising directly out of personal experience not shared more widely. As a form of distinctly discursive relevance-seeking, we suggest that ‘human condition’ statements are worth further investigation.

A second, related phenomenon observed in the discussions was the conspicuous number of self-qualifying statements. These included statements like ‘I don’t know whether anyone else felt that’ or ‘maybe that’s just me’, and other indications of not knowing, not remembering, or otherwise emphasising one’s subjectivity or bias. These statements, occurring significantly more often in MT, were a frequent feature in both groups’ discussions. Although self-qualification could be seen as a trivial indicator of default false modesty, it may also serve a helpful function for social cohesion by moderating the strength of claims being made (including softening human-condition observations). Pragmatically, it often also seemed to provide an easy entry point for the next speaker:

YELLOW: *Um, yeah, but again it’s not in the same way, he’s not hating himself anymore there. He’s just indifferent to himself. It’s a different kind of stance, you know. I mean he doesn’t seem petty, whereas I think up to this point, nearly everything he did is petty, you know like the little breakdown he has, and you just you know, you wanna give him a slap and say snap out of it. But here, I felt that was — again, does anybody else have any feelings on that, cos I’m not sure…*

GOLD: *I might agree, if it wasn’t for the fact that he carried on writing.*

YELLOW: *Yeah, fair point.* (MT-S5)

Self-qualification may also, rather than being a causal driver in its own right, be an effect or a correlate of other ways in which discussion is made more inclusive, for example a general awareness of the importance of maintaining conversational flow amongst participants. In this sense it may relate to the various form of semantic and syntactic echoing identified as a significant contributor to positive group dynamics by other researchers ^5, 11^.

Finally, VAD-structured word clouds provide a different type of clue to textual manifestations of constructive conversational dynamics. For example, the word *solution* was the most frequently spoken in the high-valence and high-dominance selections for HT ( Figure 5), having arisen 13 times in S4 (and almost never in any other session), which was the highest-dominance and highest-valence of all 6 sessions. Instances of its use make clear that participants were grappling with the difficulties of trying to make sense of the plot and authorial intention (in this instance, examples of closed complexity), as well as its wider relevance to problems humankind are currently facing (e.g. overpopulation), with more open-ended scope. Throughout the discussion, the word *solution* operates as a fulcrum between exploration of closed and more open forms of complexity. Its usage patterns add detail to the suggestion that such transitional dynamics may be associated with positive valence and feelings of control over the discussion. Thus VAD-structured word frequency mapping may, alongside human-condition observations and self-qualifying statements, be a useful indicator as to where to embark on close reading as a source of more sensitive insights regarding the direction taken by a specific text-prompted discussion.

### Transcending the qual-quant distinction

Though our study was motivated primarily by the desire to observe the effects of group reading, we feel that some concluding comments are in order on the methods that we used to conduct our analysis. In particular, we made a conscious effort to transcend the too-easy distinction between qualitative and quantitative methods. For good reason, work on literary texts has traditionally been qualitative in nature; texts, after all, are vehicles for creating meaning, and meaning can be experienced but not enumerated. Moreover, for much of the history of literary studies, computational methods simply did not exist (or exist with sufficient availability) to be used in the course of routine scholarship. This situation has changed markedly in the last decade or so, and the emergence of text embeddings and large language models has conclusively demonstrated that semantic content can be captured numerically. This means that the insights generated by contextually informed close reading can now be complemented by reliable and accessible methods from NLP and computational linguistics. 

As with all interdisciplinary undertakings, the results may not cohere in any absolute way, or they may cohere at levels other than those expected. Nevertheless, only a curiously shortsighted view of textual scholarship would reject the potential offered by the triangulation of qualitative and quantitative approaches to language. Our aim here has been to demonstrate one way in which such complementarity might play out; there are many others. But whichever one is chosen, we believe that the project of understanding how texts, culture, and cognition interact will be furthered by using all the methodological tools available––and that rejecting qualitative tools in favour of quantitative ones, or vice versa, is merely to dress up subjective prejudice as intellectual conviction. 

### Taking the longer view

A general question underlying the analyses in this study is a question about timescales. In particular, is it possible (or indeed likely) that a reading and discussion experience that has negative qualities (feels unpleasant, uncomfortable, or even unsafe) may elicit positive change (increased understanding, constructive action, etc.) at some point following the reading and discussion? Conversely, are the most positive experiences (on whichever metrics we select) more or less likely to generate positive change (of whichever type is valued)? This question of short- versus longer-term good versus ill is one that we addressed through in-depth analysis of the verbal dynamics of the discussion sessions and consideration of the participant feedback at the end of each group’s series of meetings. The free-response feedback from both groups included observations on a) learning about oneself and others, b) mood/relaxation benefits, and c) changed habits or attitudes around reading that cannot necessarily be gleaned from analysis of what was said during the discussions, and particularly not from the levels of reported enjoyment of the texts being read. Here is a selection of the concrete changes reported:

*I learned that it is hard for me to clearly articulate certain types of emotional or experiential responses and maybe that is something I need to work to improve!*

*I’m actively looking for a book to read in its entirety. How long this motivation will linger... I’m not sure.*

*I emerged with a sense that people are nicer than I thought they were, and more inclined to be charitable in my interpretation of motives more generally.*

*I became more relaxed with respect to other problems after the reading group.*

                                                                                                                                                                                          (MT)

*it has thankfully increased willingness to see others points of view.*

*I came back in a much better mood. I would listen to my daughter when we were reading together and discuss what was happening.*

*I built a momentum as it were, continuing with the state of free flowing self expression even after the session.*

*You realise who you may look and sound alike, have similarities with and it provided a new talking point(s), idea, concept to recommend to others (without discussing content of discussions).*

*It’s been a busy and sometimes difficult term for me since recovering from surgery- it’s one of several activities that I’m pleased I took part in and proud I completed.*

*Yes wanted to be able to have a new routine and read around topics not necessarily thought of or enjoyed before. Also to further number of folk we know.*

                                                                                                                                                                                          (HT)

It may or may not ever be possible to predict on the basis of textual and/or discussion content which of these benefits is mostly likely to accrue, let alone to tailor text, select participants, or guide discussion to maximize its likelihood. We suggest the next steps for group bibliotherapy research and practice should be open to several possibilities:

1) that negative effects are possible, in the short and/or longer term

In two personal reports which ET made after HT-S5 and HT-S6, she reports feeling ‘ *excluded*’, feeling that ‘ *there was no space and no time for me in the conversation*’, feeling resentment of other participants for never allowing silence between contributions, being keen to get away after the end of the session, finding it hard to re-engage with people after the end of the session, and subsequently feeling ‘ *distanced from everything and everyone, and angry, and very fragile*’ for a number of hours, including responding to mental illness-related online material in a ‘ *more viscerally defensive/aggressive*’ mode than usual. She concludes: ‘ *I’m left feeling that in the wrong hands, or with the ‘wrong’ text, or just through bad luck, this thing really could be dangerous for people. I’m generally tired at the moment, but I’m healthy and generally strong and fairly well-balanced. If I weren’t, I imagine that getting past this might have taken much longer. And who knows, perhaps with more time still, I’ll feel that I’ve learned something about myself that needed to be learned, and that the negative short-term reaction was a fine price to pay for insight that took longer to come. But right now, I feel dislike and resentment and a lingering sense of unsettlement that I can’t quite see turning into something good.*’

2) that enjoyment of the discussion may be minimally related to liking of the text

*I’ve learnt that I can still enjoy reading a text I don’t like when it’s in this kind of context.* (HT post-participation feedback)

3) that positive and negative effects of participation may be minimally related to liking of the text

*My relationship with reading has definitely changed. I see the value that it has to create a new and sometimes uncomfortable experience. Previously, I only considered reading to be strictly for the purpose of gaining knowledge. I also feel confident in sharing my opinions about a book.* (MT post-participation feedback)

4) that positive effects of participation may be related to the text-proximity of the discussion

*I also learned how reading a work of fiction together can bond people and allow for the development of group identity. I felt that reading in a group fostered a sense of collaborative journeying through the text, with the acts of reading and discussion marking a unique space and time.* (MT post-participation feedback)

The last two quotations also indicate the value of experiencing a text for the first time in the bibliotherapeutic group, as well as the significant felt impact of discovering it together. Reading aloud, as a process that heightens the experience of a shared and ‘live’ sensory-cognitive journey, is likely to be relevant here. In other words, the way a text is encountered matters, possibly more than the nature of the text itself. As for the group discussion, having begun with a sceptical attitude as to the value of actually talking about a book as opposed to simply convening for discussion about something else, we find ourselves concluding that the book really does make a huge difference. In particular, our results suggest that talking about the book is importantly different from not talking about the book, and that emotional unpredictability is greater when the book is left behind. *Literary scholars persuaded that literature has effects* is not the world’s most attention-grabbing headline, but we hope that this pilot study has demonstrated how novel quantitative methods for sensitive textual analysis can flesh out the claim and generate useful hypotheses for future testing.

## Data Availability

Oxford University Research Archive: Books, Minds, and Bodies dataset. https://doi.org/10.5287/bodleian:gJZz9KDE0 ^56^. This project contains the following underlying data: Qualitative_coding_results.xlsx raw_text_data.xlsx text_sim.xlsx qualitative_coding_results.xlsx participant_feedback_results.xlsx Data are available under the terms of the Creative Commons Zero "No rights reserved" data waiver (CC0 1.0 Public domain dedication).
