# Supplementary material for: Quantitative methods for group bibliotherapy research: a pilot study
Source: Wellcome Open Res. 2024 Jan 4;7:79. Originally published 2022 Mar 7. [Version 2] doi: 10.12688/wellcomeopenres.17469.2 (PMC10905136; doi:10.12688/wellcomeopenres.17469.2)
Supplement: Peer review history

# Reviewer report: Reviewer response for version 2

**DOI:** 10.21956/wellcomeopenres.22712.r72253

**Published:** 2024-02-28

**Authors:** Moniek M. Kuijpers

**Affiliations:** 1 Universitat Basel, Basel, Basel-Stadt, Switzerland

The authors have thoroughly revised the paper based on my feedback and that of the other reviewer. I am happy to say I approve of this version of the paper as it is without any reservations. It was a pleasure reading it.

Is the work clearly and accurately presented and does it cite the current literature?

Yes

If applicable, is the statistical analysis and its interpretation appropriate?

Partly

Are all the source data underlying the results available to ensure full reproducibility?

Yes

Is the study design appropriate and is the work technically sound?

Yes

Are the conclusions drawn adequately supported by the results?

Partly

Are sufficient details of methods and analysis provided to allow replication by others?

Yes

Reviewer Expertise:

Empirical literary studies; narrative absorption; shared reading and well-being; digital social reading; psychometrics

I confirm that I have read this submission and believe that I have an appropriate level of expertise to confirm that it is of an acceptable scientific standard.

# Reviewer report: Reviewer response for version 2

**DOI:** 10.21956/wellcomeopenres.22712.r72254

**Published:** 2024-02-02

**Authors:** Thor Magnus Tangerås

**Affiliations:** 1 Westerdals School of Communication, Kristiania University College, Oslo, Norway

I have now carefully read the article. I am quite impressed with the authors rigorous and extensive revisions, they are both appropriate and sufficient. Thus I find that the article can now be unconditionally approved.

Is the work clearly and accurately presented and does it cite the current literature?

Yes

If applicable, is the statistical analysis and its interpretation appropriate?

I cannot comment. A qualified statistician is required.

Are all the source data underlying the results available to ensure full reproducibility?

Yes

Is the study design appropriate and is the work technically sound?

Yes

Are the conclusions drawn adequately supported by the results?

Partly

Are sufficient details of methods and analysis provided to allow replication by others?

Yes

Reviewer Expertise:

Transformative reading experiences, shared reading, bibliotherapy

I confirm that I have read this submission and believe that I have an appropriate level of expertise to confirm that it is of an acceptable scientific standard.

# Reviewer report: Reviewer response for version 1

**DOI:** 10.21956/wellcomeopenres.19317.r56785

**Published:** 2023-05-24

**Authors:** Moniek M. Kuijpers

**Affiliations:** 1 Universitat Basel, Basel, Basel-Stadt, Switzerland

This article introduces newly developed quantitative measures that can be used in the context of group or shared reading to evaluate the fluctuation and variation of emotional and cognitive aspects both in the texts that are read, and the discussions that follow these readings. These tools could be used to investigate whether and how textual features in the group discussion match textual features in the text that is read, allowing for more clearly pinpointing the mechanisms underlying group or shared reading that lead to various effects (such as therapeutic outcomes).

 The article is written well, provides a clear and valuable contribution to the field and the discussion of the benefits of shared or group reading. I especially appreciate the critical reflection on bibliotherapy research, as this is something that is rarely touched upon in the literature, but is something that deserves our attention. We need more research in this area that is (as) free (as possible) of researcher bias. 

 I do have a couple of concerns I share with the first reviewer. Addressing these would certainly strengthen the article. Most notably, I agree with the first reviewer that a clearer distinction should be made between The Reader's intervention Shared Reading and the group reading that was performed in this study. As the other reviewer suggests, you have basically invented and tested your own intervention, which I do not see as problematic in itself. However, I do see the need for reflecting more on the differences between your intervention and the Shared Reading intervention (mainly in the discussion section of your paper). Especially, with regard to facilitation and text selection.

 In my understanding, Shared Reading does not involve reading out loud by all participants, just by a trained Reader Leader, which I think changes the directions in which the discussions are flowing, as compared to your intervention where the facilitator role is being shared between participants. With respect to the reflection at the end of the article about "if being left in the wrong hands, or with the wrong text, this thing could really be dangerous for people", I think this is a crucial difference to be discussed.

 With regard to text selection, I found it interesting that you found that the text selection did not seem to matter much to your participants in terms of enjoyment of discussion or overall positive or negative effects of participation, as the text selection is a major tenant of the Shared Reading intervention. Additionally, you found that engagement with the language of the text was deemed of low significance for your participants. I was hoping to see more of a reflection on these results in the discussion, especially in relation to the Shared Reading intervention's emphasis on using literary texts of "high quality".

**Methods**

 I also agree with the first reviewer that you will have to distinguish better between the two different methodological layers of your study. I would advise you also to make these layers clearer in your title and abstract, as the observational and qualitative coding you performed seem to me to be at least just as important and relevant (with respect to the main findings in your discussion) as the quantitative methods you employed.

 Where I disagree with the first reviewer is the section on the tried VAD and doc2vec methods. To me the rationale for developing these methods was sound, as was much of the explication. Where I lost the "plot", was in how the method was applied.

 I think the idea of mapping the emotional and cognitive variability in both the literary texts read and the transcripts of the discussions they inspired is a great idea, which is why I did not understand concatenating the data from the different sessions, in which you read different texts. And generally I had a hard time understanding why you would like to work with means, rather than with the variance your data shows per session (and use that as a way of generating hypotheses about differences between why certain texts lead to certain discussions). For example, the two wordclouds in Figure 5 almost look identical, which made me question what the usefulness of the method is, when collating data over several sessions.

 Furthermore, I think you described the main purpose of introducing such methods into this field of study very well in your discussion, when you say: "VAD-structured word frequency mapping may, alongside human-condition observations and self-qualifying statements, be a useful indicator as to where to embark on close reading as a source of more sensitive insights regarding the direction taken by specific text-prompted discussion". What I take from this is that it is one method that should be combined with other - more qualitative - methods to help researchers determine where in there large amount of textual data they should look for insights into "how group reading works". I think it would improve the legibility of the paper, as well as the contextualization of this method in the larger research area, if you mention something like this earlier in the paper. And generally emphasize the importance of "method triangulation" in this field of research: yes, it is important to develop quantitative methods to investigate shared reading, but they should still be combined with qualitative data to make sense of what is happening during shared reading. The methods complement each other. 

**Results**

 Could you add a laymen terms explanation to the result that text-discussion similarity was inversely correlated to emotional volatility in group discussions? Does that mean that when there was more similarity between the words used in the text and the words used in the discussion of that text, there was less volatility in those discussions? And what exactly does volatility refer to (how should we contextualize it in the VAD context)?

 I am personally more used to seeing valence  operationalized as two distinct categories: positive versus negative valence. How should we interpret valence in your study? When valence is high, does that mean that words are more pleasurable or more unpleasant?

**Discussion**

 When you discuss doc2vec similarity on page 16, I get the impression that you consider it the same thing as perceived similarity between reader and character. Am I right in assuming that, based on this paragraph, and if yes, could you elaborate on why you think it is similar? I get the sense that the doc2vec method can tell us something about the semantic similarity in terms of what words are used in the texts that are compared, but whether it could really help us reflect on the perceived similarity that readers felt between them and the characters in the story, I am unsure. Of course, you can use your observations and coding here to reflect on the importance of perceived similarity.

 In general I would really like to see more of a reflection on the usefulness of the newly developed quantitative methods in the discussion. Right now, the discussion is mostly dedicated to - really important and relevant - insights gleamed from the observational and qualitative coding methods used, whereas the title of the paper would make the reader assume it is mostly about the quantitative methods.

**Minor points**

 These are some smaller suggestions to improve the overall legibility of the paper.

- I would consider changing the abbreviations HT and MT into something more general like ST (spring term) and FT (fall term), so it is easier for other researchers to interpret (those of us that are not used to the specific terminology used at Oxford University)
- Related to that, sometimes when you are drawing conclusions about differences between the MT and the HT group it is unclear whether you are referring to differences between the groups themselves or differences between gathering during spring term versus fall term (e.g., greater emotional febrility in HT)
- Could you elaborate what the column "Commentary" refers to in Table 2.
- The first part of the note under Figure 5 does not seem to correspond to the actual figure (Figure 5 is just about the HT group, whereas the note implies that it is about both groups)

Is the work clearly and accurately presented and does it cite the current literature?

Yes

If applicable, is the statistical analysis and its interpretation appropriate?

Partly

Are all the source data underlying the results available to ensure full reproducibility?

Yes

Is the study design appropriate and is the work technically sound?

Yes

Are the conclusions drawn adequately supported by the results?

Partly

Are sufficient details of methods and analysis provided to allow replication by others?

Yes

Reviewer Expertise:

Empirical literary studies; narrative absorption; shared reading and well-being; digital social reading; psychometrics

I confirm that I have read this submission and believe that I have an appropriate level of expertise to confirm that it is of an acceptable scientific standard, however I have significant reservations, as outlined above.

# Response

**Published:** 2023-12-21

**Authors:** James Carney

**Affiliations:** Brunel University London, UK

**Response to Reviewer 2** **Introduction/general** Thanks for these comments on the overall thrust of the paper, particularly as regards the distinction with Shared Reading. We have included more detail on the specific differences between our procedures and SR (with respect to facilitation, reading aloud, and text selection) and have also flagged more clearly that this is in fact a new set of procedures. We added a note near the end on the link between facilitation procedures and harm reduction. With respect to text selection, we added further arguments to justify our approach: specifically, that our texts needed to respect the previous reading of the participants, and were by any reasonable metric of a high literary quality. With that said, we also added the point that judgements of literary quality are not stable over time and individuals, and there will inevitably be a large amount of subjectivity in any text selection. **Methods** We clarified some points where our methods were not clear with respect to concatenating sessions together (which we didn’t actually do) and justified our decision to use point estimate measures (means) rather than measures of spread (variances). We also corrected the incorrect word cloud figure that you helpfully pointed out.   We appreciate your comment on our mixed methods, and we’re glad that you consider the qualitative coding to add value. On balance we feel that the computational methods trialled here represent the methodological innovation and thus are appropriate to foreground in the title. We have added mentions of the qualitative analysis and other contributions from the Discussion section to the abstract to give them more salience. And see below for an addition to the Discussion section itself regarding the important principle of quant/qual triangulation. **Results** We added some text that clarified what doc2vec, which we used to measure semantic similarity, can and cannot measure with respect to reader response, and briefly clarified our use of the term “emotional volatility”. Our use of a scalar (rather than polar) measure of valence was also justified. **Discussion** Expanding on our elucidations of the doc2vec method noted above, we also added clarification of its relation (or lack of) to reader/character similarity. We chose to dedicate a good proportion of the Discussion section to unpacking the insights gleaned from the qualitative analysis, as a way to conclude the paper on a more exploratory note and give the qual elements meaningful space. But in the effort to connect the two sets of methods more satisfyingly with each other, we have added a short new section on the significance of the quantitative methods and on the triangulation attempted here between the two. **Minor points** We considered various alternatives to the (admittedly arcane!) Michaelmas and Hilary, but after reflecting that no alternatives are problem-free (autumn versus fall; spring versus winter term; etc.) we opted to let MT and HT stand.   In all cases, mentions of intergroup differences refer to differences observed between the two groups as run in the two terms. We were unable to identify simple ways to clarify this further in the main text, but we hope this note provides adequate clarification. The somewhat cryptic Table 2 column label has been clarified.  And thanks again for spotting the incorrect file inclusion in Fig. 5. Thanks for your thorough and helpful feedback!

# Reviewer report: Reviewer response for version 1

**DOI:** 10.21956/wellcomeopenres.19317.r49064

**Published:** 2022-04-04

**Authors:** Thor Magnus Tangerås

**Affiliations:** 1 Westerdals School of Communication, Kristiania University College, Oslo, Norway

The article is interesting and provides a valuable contribution to research on literary reading in groups and the development of quantitative methods for analysing emotional and cognitive aspects of text-discussion relationships and complex reader responses.

 The article is clearly structured, well written and engaging. The introduction covers most, but not all (see further down), relevant research on the effects and mechanisms of various bibliotherapies.

 The rationale and objective of the study is clearly laid out:

 There is an absence of analytical methods capable of providing sensitive yet replicable insights into complex textual material.

 This pilot study offers a proof-of-concept for new quantitative methods including VAD (valence–arousal–dominance) modelling of emotional variance and doc2vec modelling of linguistic similarity.

 And the sections on method, results and discussion are thorough and transparent. The analytic method is replicable.

 In sum, I recommend that the article be indexed, but there are issues that should be addressed in each section of the paper:

**Introduction:**

 The article would profit from a sharper discussion of what is implied by “bibliotherapy”.

 At least two things should be highlighted here:

- That “bibliotherapy” is a contentious term, and covers a highly diverse set of practices, contexts and rationales. For instance, some forms are centered around an interview with a prospective reader with a problem, the problem is analysed and the “bibliotherapist” selects and recommends apt reading material. What constitutes “apt” literature is problematic. E.g. should choice of text be based on “similarity of problems”? And the current study provides empirical findings of great value here.
- Shared Reading, the group reading practice that is now most widely disseminated (not just in the UK but across Scandinavia and Europe) does not call itself a “bibliotherapy”. The purpose of Shared Reading was to allow everyone regardless of background the access to and enjoyment of “great reading” (cf Jane Davis, The Lancet). Therapeutic effects were seen as secondary gains, and not the effect aimed for. I think the current article would do well to recognize that a central premise of SR is that the chosen literature may not be “likeable” – challenging, threatening, etc. As such, this study lends empirical support to this central premise.

Also, a little bit of historical context would be useful. The “theorization” of bibliotherapy consists largely of constructs borrowed from psychoanalysis (identification, catharsis, etc). It is only in recent years that empirical approaches to bibliotherapy and literary reading as such have gained currency. And the most relevant of this research – that carried out by Kuiken *et al. *on the one hand, and Phil Davis *et al.* – is primarily bottom-up and not theory driven.

 Given that the article briefly discusses Billington’s account of four “mechanisms”, and the problems associated with establishing the impact of each, it would be useful to discuss how

 One could isolate variables, given that this study in fact revolves around discussing the role of two of these: choice of reading material and group discussion, but has altered one premise of SR – that the facilitator be trained and highly skilled.

**Method:**

 What is referred to as “method” here (and occasionally also “methodology”) is problematic.

 On the one hand, it comprises reading group procedures and participants, on the other the methods of analysing data.

 What should be stated clearly is that the authors have in fact invented their own group reading method. Whether that should be called Bibliotherapy or something else is another matter. Table 1, designed to show the differences in variables among various approaches, does not cover all relevant aspects here.

 First of all, why have the authors decided to design their own unique blend? Given that Shared Reading forms the central touch point in terms of empirically-based group methods, perhaps it would be better to gather data from SR sessions? If not, why not?

 Very little is said about the participants. Are they all academics/students at Oxford? Are they all avid readers? They are interviewed beforehand and primed as to their role, so they are highly motivated to participate. This is relevant. They get to choose the reading material, but from which options? And based on what? Perhaps the issue of identification or likeability or plot interest is already integral to that choice.

 Why not discuss the relevant differences to Shared Reading? I think it is significant that the session is divided in two here: first read, then discuss. But the most problematic aspect is that facilitation is rotated among participants. The list of possible questions they are given beforehand are mostly evaluative, asking for a “head” response rather than immediate emotional reactions as they happen. This is a very important objection given that so much of the discussion of the results is about emotional responses.

 Method of analysis:

 This part is the central part of the method. How can one find and develop quantifiable and replicable ways of determining emotional and cognitive responses and interactions?

 I find the explication of rationale  for selecting VAD and doc2vec, and the procedures and documentation of their implementation, solid and convincing. This part of the article needs no alterations. What is worth discussing, however, given that this is a pilot study, is: what would the difference be if a different emotion model was used?

**Results and Discussion:**

 Results are clearly laid out and analysed. Emotions by session, by literary text, by word norm data – and cognitive elaboration.

 The discussion is relevant and thorough.

 My main objective here is:

 There is a significant problem in that the article does not clearly distinguish between reader responses, interactions and reported worth of participation on the one hand, and therapeutic effects on the other. Cognitive and emotional engagement with text and with group can be established, and also how the participants self-report the value of participating. However, there is no bridge to documentation of therapeutic benefits/improved psychological well-being over time.

 As stated in the article: Our analysis used new quantitative methods in the attempt to provide a combination of richness and replicability needed to answer questions about human responses to complex aes- thetic phenomena.

 There is a big difference between the question of emotional-cognitive responses to aesthetic phenomena and the question of the therapeutic benefits of these responses. As such, the findings of the study may be more relevant to the discussion of reader response theory than bibliotherapy theory.

 In the literature review, some highly relevant studies are omitted:

 Longden, E., Davis, P., Billington, J., Lampropoulou, S., Farrington, G., Magee, F., ... Rhiannon, C. (2015). Shared reading: Assessing the intrinsic value of a literature-based health intervention. Med Humanities, 41, 113–120. doi: 10.1136/medhum- 2015-010704. ^1^

 Longden, E., Davis, P., Carroll, J., & Billington, J. (2016). An evaluation of shared reading groups for adults living with dementia: Preliminary findings. Journal of Public Health, 15(2), 75–82. doi: 10.1108/JPMH-06-2015-0023. ^2^

 One of these studies does in fact have a control group where a different group activity is used. And in one of these studies, pre-/post testing is used. If objective/quantifiable measures of improved mental health/wellbeing are to be found, there must be a concept of wellbeing and a way of measuring it systematically. Thus, if it were shown that participants did improve mental health, then hypothesizing that this improvement would be reflected in the transcripts would be of great importance.

 (Another article not cited, (Shared reading as an affordance-nest for developing kinesic engagement with poetry: A case study, Kjell Ivar Skjerdingstad and Thor Magnus Tangerås) Discusses how participation can be of benefit even when one does not like the text.) ^3^

 In sum, the article would profit from:

- Clearer context for study.
- Mention Longden *et al.*s articles.
- Discussion of rationale and choice of procedure of group method.
- In the discussion section, draw a distinction between therapeutic effects and reader responses. And elaborate on how the method can be developed further, and which hypotheses can be tested and which variable must be controlled for

Is the work clearly and accurately presented and does it cite the current literature?

Yes

If applicable, is the statistical analysis and its interpretation appropriate?

I cannot comment. A qualified statistician is required.

Are all the source data underlying the results available to ensure full reproducibility?

Yes

Is the study design appropriate and is the work technically sound?

Yes

Are the conclusions drawn adequately supported by the results?

Partly

Are sufficient details of methods and analysis provided to allow replication by others?

Yes

Reviewer Expertise:

Transformative reading experiences, shared reading, bibliotherapy

I confirm that I have read this submission and believe that I have an appropriate level of expertise to confirm that it is of an acceptable scientific standard, however I have significant reservations, as outlined above.

## References

[1] : Shared Reading: assessing the intrinsic value of a literature-based health intervention. *Med Humanit* .2015;41(2) : 10.1136/medhum-2015-010704 113-20 10.1136/medhum-2015-010704 26070845

[2] : An evaluation of shared reading groups for adults living with dementia: preliminary findings. *Journal of Public Mental Health* .2016;15(2) : 10.1108/JPMH-06-2015-0023 75-82 10.1108/JPMH-06-2015-0023

[3] : Shared reading as an affordance-nest for developing kinesic engagement with poetry: A case study. *Cogent Arts & Humanities* .2019;6(1) : 10.1080/23311983.2019.1688631 10.1080/23311983.2019.1688631

# Response

**Published:** 2023-12-21

**Authors:** James Carney

**Affiliations:** Brunel University London, UK

**Response to Reviewer 1** **Introduction** ****Thank you for your helpful pointers to fleshing out our account of bibliotherapy, emphasising the breadth and contentiousness of the term and in particular its uneasy relationship with the Shared Reading paradigm. The Davis reference is very helpful there. We’ve made some additions to the Introduction to reflect these tensions, and have added something more explicit on why we do choose to use the term in the group reading context here. We appreciate the point that SR’s take on “likeability” is neatly compatible with our conclusions here about liking and enjoyment, and have drawn out that link explicitly. And we’ve made a few small additions on the history of bibliotherapy as theory and practice; thanks for the helpful sketch of the main contours. It’s a nice point that our study works directly with two of Billington’s four hypothesised mechanisms while testing the counterhypothesis with the trained facilitation. We added a brief note on testability, including in relation to the structure of our study. **Method** We understand that foregrounding “methods” in the study title makes any terminological slippage here particularly salient. Overall, the scientific convention in which “Methods” covers all practices, both analytical and procedural, has contributed to some fuzzy boundaries here, but we have replaced “methods” with “procedures” where appropriate, to distinguish more clearly between the reading-group setup and the data analysis.  Thanks also for the suggestion to demarcate our reading-group procedures much more explicitly from the SR procedures. We now have a section enumerating the major differences and offering a rationale for adopting these rather than the typical SR methods—allowing that there is already variation amongst SR implementations, as shown in Table 1 (which has also been adjusted slightly to offer a more informative overview). We have now included a little more detail on the participants’ backgrounds, the initial interview, and the text selection process (touching also on the likeability question). On the last of these, the shortlist choices were inevitably arbitrary to some extent, but we did ensure that no single person’s taste or judgement predominated.  As for facilitation procedures: We have attempted to clarify in the main text that the facilitation questions offer, in our view, a reasonable balance of interpretive, emotional, sensory, and personal-relevance aspects, while being constrained by the fact of being precisely a retrospective discussion rather than attempting to tap in-the-moment immediacy. The latter would require significantly different methods (more along the lines of ecological momentary assessment, for example—which would be tricky to implement in a group setting). Never having taken part in a SR session ourselves, we don’t know how the decisions about when to pause to discuss—or indeed how to guide discussion in one direction or another—are in practice taken, in the training protocols or in their translation to specific groups. More investigation of what difference these small differences make would be an interesting direction for future research. **Method of analysis** We have clarified and expanded on our methods in several places where we were not sufficiently clear in our original text. We explained why the VAD model was used instead of other models of dimensions of emotion, and why we expect (though cannot prove) that there would be no substantive differences in our results if we had VAD-analogous linguistic data for other models of emotion. **Results and Discussion** We went to greater lengths to clarify why the distinction between “therapeutic” and “cognitive/emotional” effects is less relevant than it first appears. We respect the reviewer’s observation that these are distinct phenomena, but we also referenced innovations in thinking about mental health that intentionally set out to collapse these distinctions in a way that is both productive and relevant to our ambitions. Thank you for flagging these three really interesting and valuable studies, all of which we have woven into our literature review, and mentioned elsewhere as appropriate. With respect to using a control-group methodology, we made it clearer that we are not engaged in hypothesis testing; our study was observational. This means that using a control vs experimental group design would have been premature. Many thanks for your constructive comments.
